# A numerical assessment of social distancing of preventing airborne transmission of COVID-19 during different breathing and coughing processes

**DOI:** 10.1038/s41598-021-88645-2

**Published:** 2021-05-03

**Authors:** Alibek Issakhov, Yeldos Zhandaulet, Perizat Omarova, Aidana Alimbek, Aliya Borsikbayeva, Ardak Mustafayeva

**Affiliations:** 1 al-Farabi Kazakh National University, Almaty, Republic of Kazakhstan; 2 Kazakh British Technical University, Almaty, Republic of Kazakhstan

**Keywords:** Computational science, Computational models

## Abstract

The spread of the novel coronavirus disease (COVID-19) continues to show that geographic barriers alone cannot contain the virus. Asymptomatic carriers play a critical role in the nature of this virus, which is rapidly escalating into a global pandemic. Asymptomatic carriers can inadvertently transmit the virus through the air stream. Many diseases can infect human bodies with tiny droplets or particles that carry various viruses and bacteria that are generated by the respiratory system of infected patients. This article presents the numerical results of the spread of droplets or particles in a room. The proposed numerical model in this work takes into account the sedimentation of particles or droplets under the action of gravitational sedimentation and transport in the room during the process of breathing and sneezing or coughing. Three different cases are numerically investigated taking into account normal breathing and coughing or sneezing, respectively, and three different rates of particle ejection from the mouth are considered. Navier–Stokes equations for incompressible flows were used to describe three-dimensional air flow inside ventilated rooms. The influence of ventilation rate on social distancing is also computationally investigated. It was found that particles can move up to 5 m with a decrease in concentration in the direction of the air flow. The conclusions made in this work show that, given the environmental conditions, the two meter social distance recommended by WHO is insufficient.

## Introduction

Since the end of 2019 and to this day the world community faced with large-scale virus SARS-CoV-2, which causes a dangerous infectious disease—COVID-19^1^. The most common complication of the disease is viral pneumonia, which can lead to acute respiratory distress syndrome and subsequent acute respiratory failure, in which oxygen therapy and respiratory support are most often needed. The most common symptoms of the disease are fever, fatigue, and dry cough. Given the rapid spread and lethal outcome of the virus, by early 2020, the infection was recognized as a pandemic and halted economic development in many countries around the world^2,3^. At the time of this paper writing, the global statistics of COVID-19 are as follows: more than 100 million people are infected, more than 2.3 million have died^3^. SARS-CoV-2, together with Severe Acute Respiratory Syndrome (SARS) and Middle East Respiratory Syndrome (MERS), is already the third highly pathogenic member of the coronavirus family over the past two decades.

The vast majority of respiratory diseases such as tuberculosis, measles, chickenpox^4,5^, influenza, bronchitis, and pneumonic plague^6–9^ are transmitted by airborne droplets. SARS-CoV-2 is also spread by airborne droplets (particles) or through close contact, according to the World Health Organization (WHO)^10^. The main mechanisms for the spread of viral diseases are coughing and sneezing.

With active breathing, sneezing and coughing, small droplets are formed, which consist of water, air, tiny particles (d_p_ < 10^–6^ m) and respiratory fluid. These components of human reflex processes have different rates and duration (time) of generation and, accordingly, lead to different effects on the environment and the human body^11–13^. The distance that these droplets can travel directly depends on the size of the droplets themselves and the speed of its propagation^14–16^. For example, a number of studies confirm that large droplets (10^–4^–10^–3^ m), due to the action of gravity, instantly settle to the ground^17,18^, while droplets with a smaller diameter size (10^–6^ to 10^–4^ m) behave similarly to gas and remain in the air for a long period of time^19^. During a 5-min conversation or with a single cough, a person sheds about 3000 microorganisms, while sneezing generates even more drops^20–22^, which are too small to be seen with the eye, but large enough to carry various respiratory pathogens^23^. Experimental studies show that coughing or sneezing, the droplet size can vary from 0.1 to 1000 μm^24–26^, which is sufficient to carry both bacteria and viruses^25^. However, it should be consider that during breathing and coughing, different droplet sizes are formed, so when breathing, small size droplets are formed, while when coughing, large size droplets are formed^27^. A study^28^ was aimed at determining the size of aerosol particles in the room, as well as studying the cough reflex in the patient. In addition, the size of microdroplets is the main factor that affects the dispersion and deposition of aerosol particles^29,30^. Since the reactive speed of coughing and breathing upon exit is on average about 1–22 m/s^26,31–33^, then the transport of exhaled drops can be conditionally divided into two stages: the primary is the jet transport during coughing/exhalation, and the second is dispersion by the air flow in the room.

Of all the innate defense reflexes, cough is probably the most studied because of its important role in disease transmission. Its high speed air flow is unstable at the interface between mucous membranes and air. Droplets formed due to the instability mechanism can be carried by the air flow over long distances after they leave the human respiratory tract^34^.

Viable influenza infectious particles are known to have been recovered by air sampling in hospitals, medical centers and aircraft^35–37^. In general, influenza viruses viable in the air from 1 to 2 h to several days^38^. It should be noted that the greatest danger is posed by localized centers of pathogenic aerosol pollutants accumulation. These surfaces include the mucous membrane of the human respiratory system, as well as the mucous membranes of the eyes, nose or mouth, which have a favorable environment for the reproduction of microorganisms. Bacteria and viruses exert a colossal effect on vital activity through carrier-to-carrier transmission^39^. In the process of transfer in this way, the smallest aerosol particles (virion) of viruses and other disease-causing particles penetrate from infected people^40–43^ to non-immune carriers. This spread occurs due to irritation of the mucous membrane of the laryngeal and nasal systems, which in turn leads to coughing and sneezing^44,45^. The rate of air exchange in a closed room (unventilated room) is limited, as a result of which it is very easy for people in this room to become infected with the virus^46–48^. With the increase in the incidence, it has become extremely important to track as accurately as possible scenarios for the behavior of viral microorganisms in an enclosed space. The nature of infectious aerosols is also greatly influenced by room ventilation, human temperature and room temperature^29,49,50^. Research results^51^ confirm that mechanical ventilation can play a role in improving overall health.

The mechanism of formation and origin of droplets with virion associated with viral and bacterial load in microdroplets because pathogens are usually limited to certain parts of the body^29^. In order to better understand the process of the formation and spread of viruses, their detailed modeling in an artificial environment is necessary^52^. Numerical simulations have also made it possible to understand the difference between sneezing and coughing from a medical point of view. Feng et al.^53^ developed a numerical model that investigates the effects of wind and relative humidity on the effectiveness of social distance to slow airborne transmission of COVID-19 disease. A computational experiment was conducted between a susceptible and coughing person with a face shield. The authors of the work^10^ numerically analyzed the sneezing process in an asymptomatic carrier of the COVID-19 viral infection, taking into account various environmental factors. Often the patient's safety against infection during hospitalization is highly questionable due to the rapid spread of contaminants and microbial droplets. In this regard^54^, carried out a numerical simulation of the air flow in a medical institution in order to obtain an acceptable indoor air quality. Xu et al.^55^ considered the area around the human face as geometry in order to simulate real air flows during inhalation and the spread of airborne particles in the room. Tracking the distribution of infectious aerosols is extremely difficult as they come in various shapes and sizes. A study^56^ demonstrated how long it takes to remove microbial droplets from sneezing from a ventilated room. The authors of the described work proposed using CFD to optimize the artificial ventilation system and remove infectious droplets. To date, there are also a series of computational experiments on sputum droplet spraying taking into account various chemical components: NaCl, amino acids, carbohydrates, and lipids that make up the droplets^57^. In the paper^58^, the authors developed a numerical model of the infectious particles diffusion at different positions of a person and at different room temperatures.

A number of studies show that the unconditioned reflexes of a person (coughing and sneezing) consist not only of muco-salivary droplets, but mainly of a multiphase turbulent gas, stratus cloud that captures the surrounding air and carries droplets with microorganisms inside it^59–61^. The theoretical model presented in^62^ characterizes the behavior of such a cloud and is confirmed by experimental data. It is shown that the turbulent dynamics of the dispersed phase is of decisive importance for increasing the range of microdroplets action due to the delay in their exit from the turbulent cloud.

Risking their lives, routine care providers work closely with patient’s suspected or even positive for COVID-19. Despite the fact that the pathogen that causes COVID-19 is not transmitted through aerosols, but through microdroplets, with a strongly directed air flow, such droplets can be carried over a distance exceeding the recommended social distance of 2 m^2,3^. It is the rapid spread of viral aerosol particles that plays a decisive role in medical institutions during outbreaks of infection. Therefore, respirators with filtering face masks have been developed to protect the medical profession^63^. Lindsley et al.^64^ assessed the minimum safe distance between a healthcare professional and a sick patient due to airborne infectious particles. From the point of view^65^, the social distance of 2 m, traditionally recommended for protection from COVID-19, does not guarantee complete safety. The social distancing policy adapted to date does not take into account the impact of relative humidity (RH)^66^.

Many infectious diseases are transmitted from person to person by inhalation of airborne droplets, which carry various viruses and bacteria that are generated by the respiratory system of infected people. People in the room can easily get the virus, which is carried by droplets or particles, because the rate of air exchange in the room is limited. Normal breathing, sneezing, or coughing produces tiny droplets of water and air or small particles. These generation methods have different generation rates and duration, which leads to different effects on the indoor environment and the human body. The main purpose of this work is to study the transport of droplets or particles generated by the respiratory system in a room during various scenarios. Since it is difficult to experimentally study the transport and diffusion of droplets or particles from the respiratory system, this work numerically investigates the spread of air droplets, sneezing and coughing in a room. Air droplets are considered small water particles.

## Mathematical model

In order to build a mathematical model of the air flow, the Navier–Stokes equations system is used, which is numerically implemented by the ANSYS Fluent 18.0. Navier–Stokes equations for incompressible cases are used to model the flow field. The continuity and momentum equations used in the model are defined as follows:

1 $$\frac{{\partial u_{j}^{{}} }}{{\partial x_{j}^{{}} }} = 0,$$

2 $$\frac{{\partial u_{i} }}{\partial t} + \frac{\partial }{{\partial x_{j} }}(u_{i} u_{j} ) = f_{i} - \frac{1}{\rho }\frac{\partial p}{{\partial x_{i} }} + \frac{\partial }{{\partial x_{j} }}\left[ {\mu_{eff} \left( {\frac{{\partial u_{i} }}{{\partial x_{j} }} + \frac{{\partial u_{j} }}{{\partial x_{i} }}} \right)} \right],$$

where $\mu_{eff}$—the effective viscosity, $p$—the pressure (Pa), $\mu_{eff} = \mu + \mu_{t}$, where $\mu_{t}$—the turbulence viscosity. The external force of the body considered is gravity, so that $f = \rho g$, where $g$ is the acceleration due to gravity (m/s^2^), $\rho$—the density (kg/m^3^).

The kinematic relationship between the position of particles (respiratory droplet) and the speed of particles (respiratory droplet) is

3 $$\frac{{dx_{p} }}{dt} = u_{p} ,$$

4 $$m_{p} \frac{{du_{p} }}{dt} = F_{D} + F_{G} ,$$

where $x_{p}$—the particles location (m), F_G_—is the gravity force, F_D_—the drag force, $u_{p}$—the velocity of particles (m/s), $u_{f}$—the velocity of fluids (m/s), $m_{p}$—the mass of particles (kg) and F_D_—calculated as follows

5 $$F_{D} = \frac{1}{2}\rho_{f} \frac{{\pi d_{p}^{2} }}{4}C_{D} (u_{f} - u_{p} )|u_{f} - u_{p} |,$$

where the resistance coefficient

6 $$C_{D} = \left\{ {\begin{array}{*{20}l} {\frac{{24}}{{{\text{Re}}}};} \hfill & {({\text{Re}} < 1)} \hfill \\ {\frac{{24}}{{{\text{Re}}}}(1 + 0.15{\text{Re}}^{{0.687}} );} \hfill & {(1 \le {\text{Re}} \le 1000)} \hfill \\ \end{array} } \right.,$$

where $\rho_{f}$ is the density of the fluid (kg/m^3^), $\rho_{p}$ is the particle density (kg/m^3^) and $d_{p}$ is the particle diameter (m), ${\text{Re}} \equiv \frac{{\rho d_{p} \left| {\vec{u} - \left. {\vec{u}_{p} } \right|} \right.}}{\mu }$ is the Reynolds number.

In order to close the system of equations, various turbulent models (k-ε, SST k-ω, DES model) were used. These turbulent models were described in detail in^67–70^. All simulations were performed using the SST k-ω turbulent model, as this model has proven to be reliable in indoor airflow modeling^71–76^.

These equations are discretized using the finite volume method. To numerically solve the system, the numerical algorithm SIMPLE (Semi-Explicit Method for Pressure Equations)^77–82, 85^ is used. This method is used in many works to solve various problems of hydrodynamics and heat transfer and has served to create a whole class of numerical methods. All variables and constants that were used in these calculations are completely physical.

## Verification

**Figure 1:**
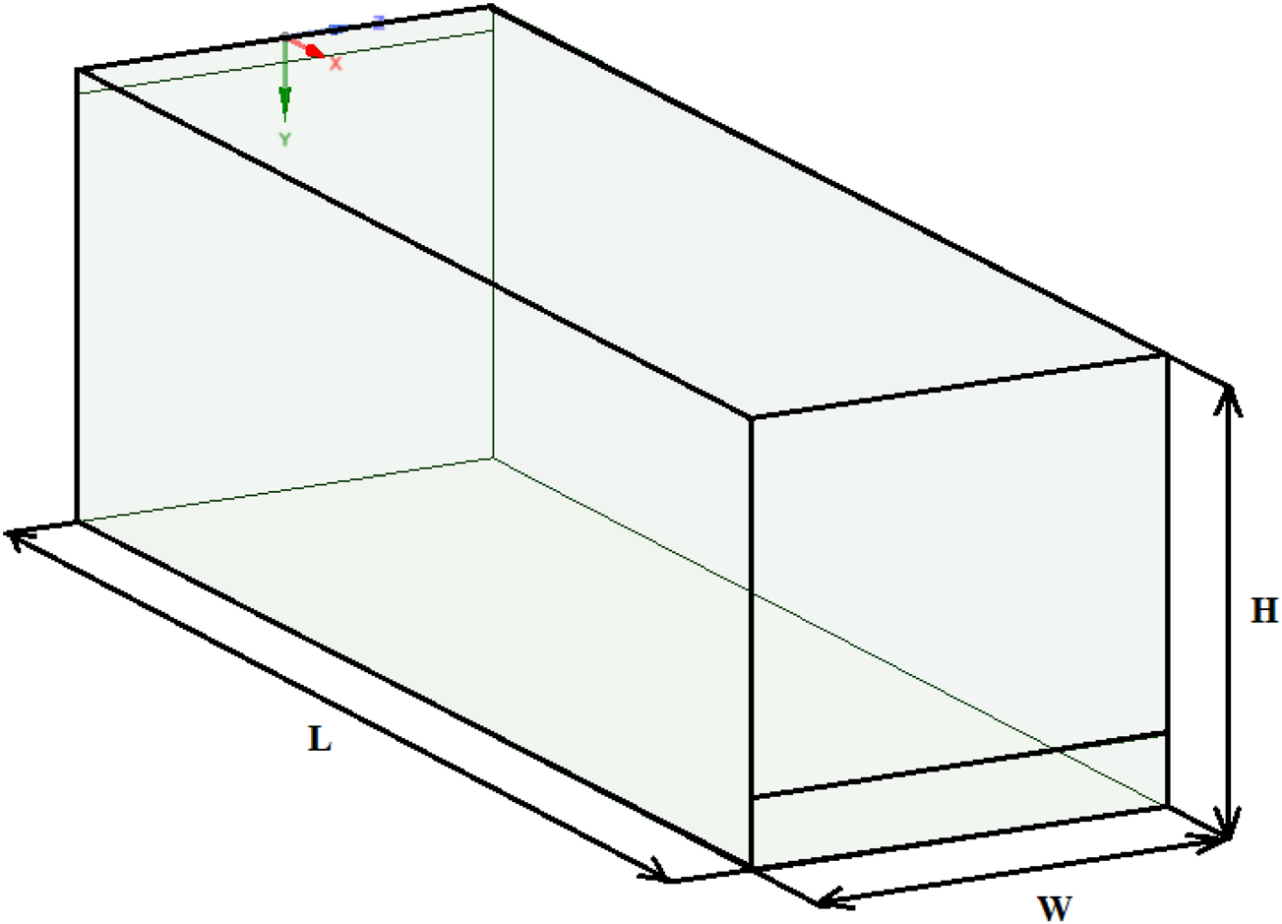
Configuration of the computational domain.

**Figure 2:**
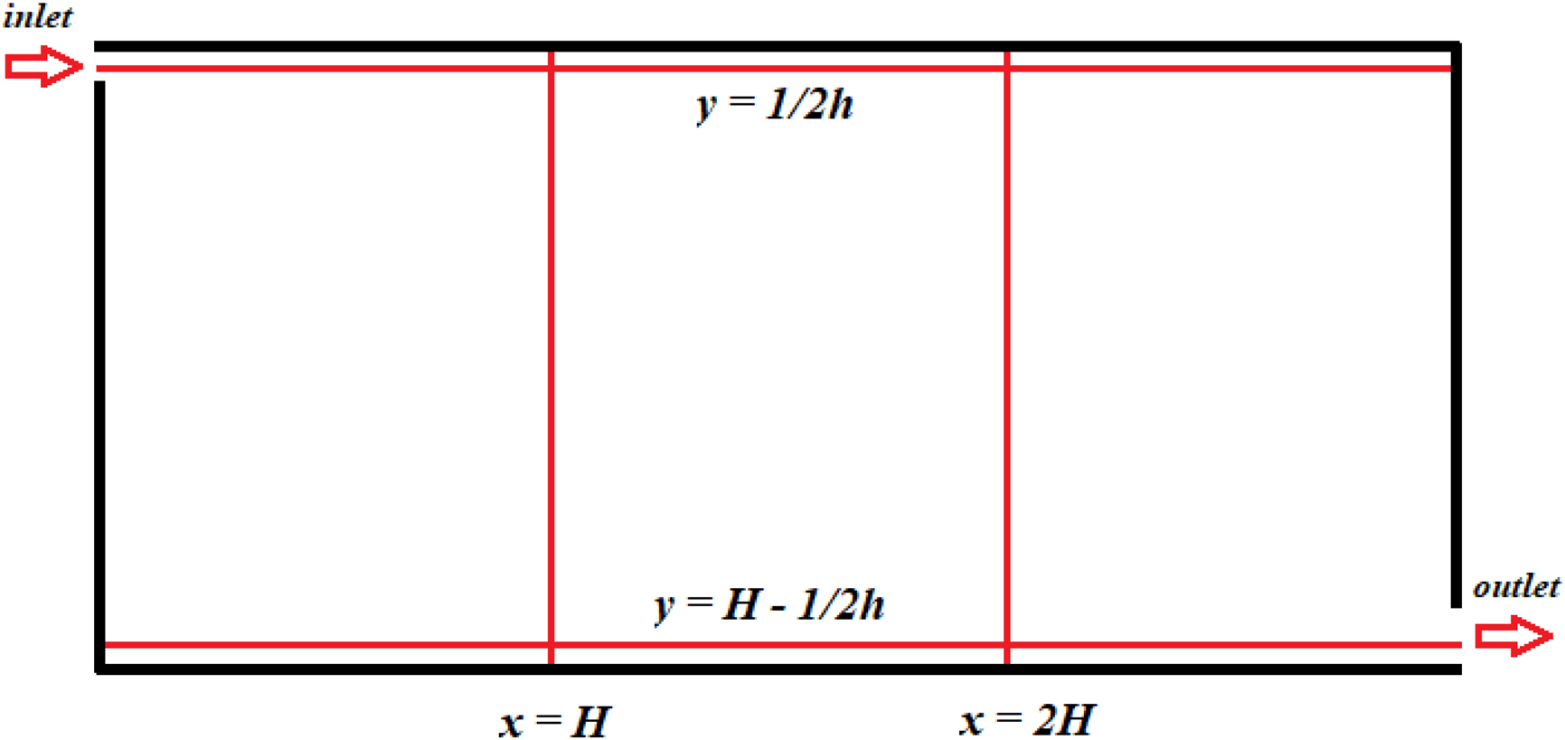
Measuring lines in the central section, z = 0.

In order to perform the test problem, a 3D model was built based on the data provided by Han et al. 2019. Experimental data obtained by Nielsen et al. was used as the basis for the test problem. The calculation area is a three-dimensional enclosed room equipped with an inlet and outlet (Figs. 1, 2).

Experimental velocity data were obtained in a room measuring 9.0 m × 3.0 m × 3.0 m (L × W × H). In this case, an entrance slit with dimensions of 3.0 m × 0.168 m (h) is installed in the upper part of the rear wall of the test chamber. An exhaust outlet measuring 3.0 m × 0.48 m is located at the bottom of the front wall.

For the simulation, the optimal computational grid was chosen, on which the most approximate solutions to the experimental data were obtained. And with a decrease in the number of elements of the computational grid, it did not greatly affect the obtained numerical solutions. To obtain an accurate result, a structured grid was built, which consists of 225 × 75 × 75 nodes, and the total number of elements was 1,171,864. For the calculation, the material at the entrance was chosen—air. For the numerical simulation of this problem, the following air parameters are taken: dynamic air viscosity is μ = 1.7894 × 10^–5^ kg/ms, density ρ = 1.225 kg/m^3^, Cp (Specific Heat)—1006.43 J/kg-K, thermal conductivity—0.0242 (W/mK), molecular weight—28.966 kg/kmol, standard state entropy—194,336 J/kgmol-K, reference temperature—298.15 K. The inlet velocity was set as $U_{in} = 0.455$ m/s. The computational experiment took 24 min.

**Figure 3:**
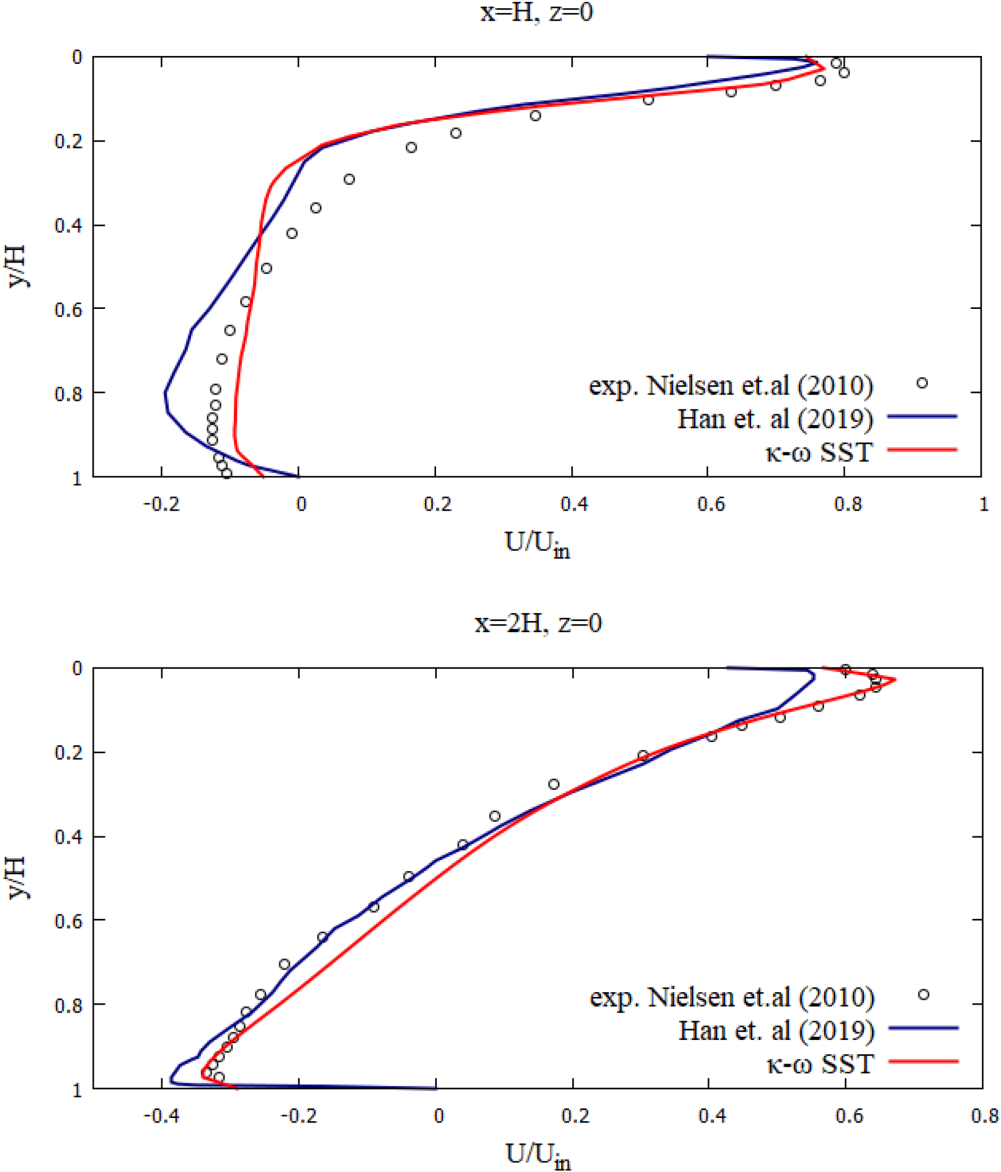

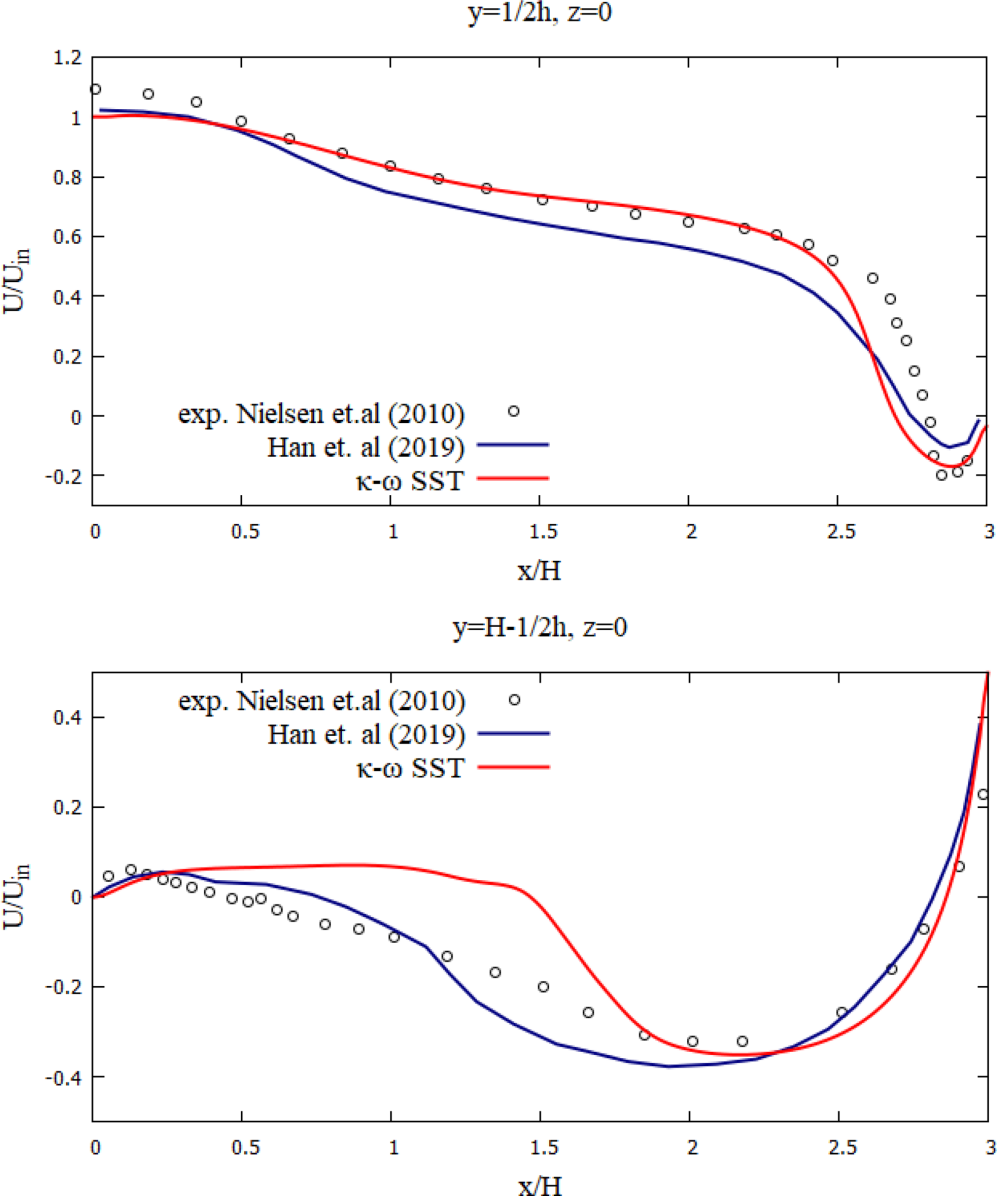
Comparison of the numerical results of the velocity profiles, with the values of the experiment by Nielsen et al.^84^ and the results of Han et al.^85^.

**Figure 4:**
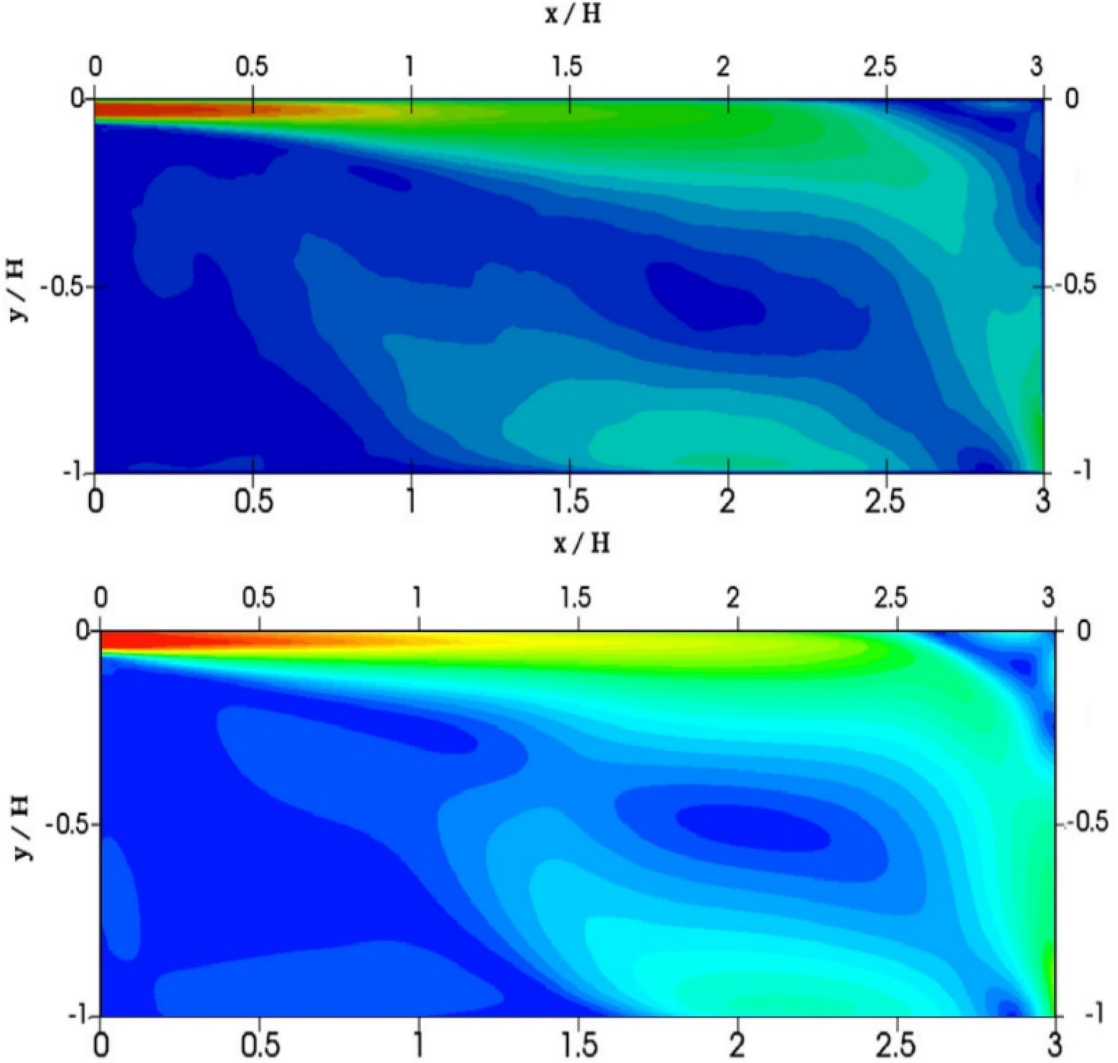
Comparison of the mean velocity contour in the central plane (z = 0): the upper figure is the result of Han et al.^85^, the lower figure is the result obtained in this work.

Figure 3 depicts the numerical results of the velocity profiles of this research work with experimental results from^84^ and the results of calculations from^85^. As shown in Fig. 3, the dimensionless velocity values obtained from this study are in good agreement with the experimental results^84^. The numerical model used can well predict the process of room ventilation. Figure 4 presents a comparative analysis of the mean velocity contour obtained in this work and the results obtained by Han et al. As can be seen from the numerical results (Figs. 3, 4), it can be concluded that the numerical algorithm proposed in this work predicts well the ventilation process in the room. As can be seen from the obtained results for some cross sections (y = 1/2 h and y = H−1/2 h), the numerical results have some deviations from the experimental data, however, the obtained solutions in this work remain the closest to the experimental data than the numerical solutions obtained by other authors^85^. These deviations can be explained by the fact that these cross-sections are at the level of the inlet and outlet boundaries and are very strongly subject to the inlet boundary conditions, so any small deviation leads to deterioration in the solution. The purpose of the test problem was to test the mathematical model for the ventilation rate circulating inside the room. This proposed model will be further used for ventilation of the room, where the process of the particles propagation emitted from the human mouth will be considered.

## Numerical simulations

**Figure 5:**
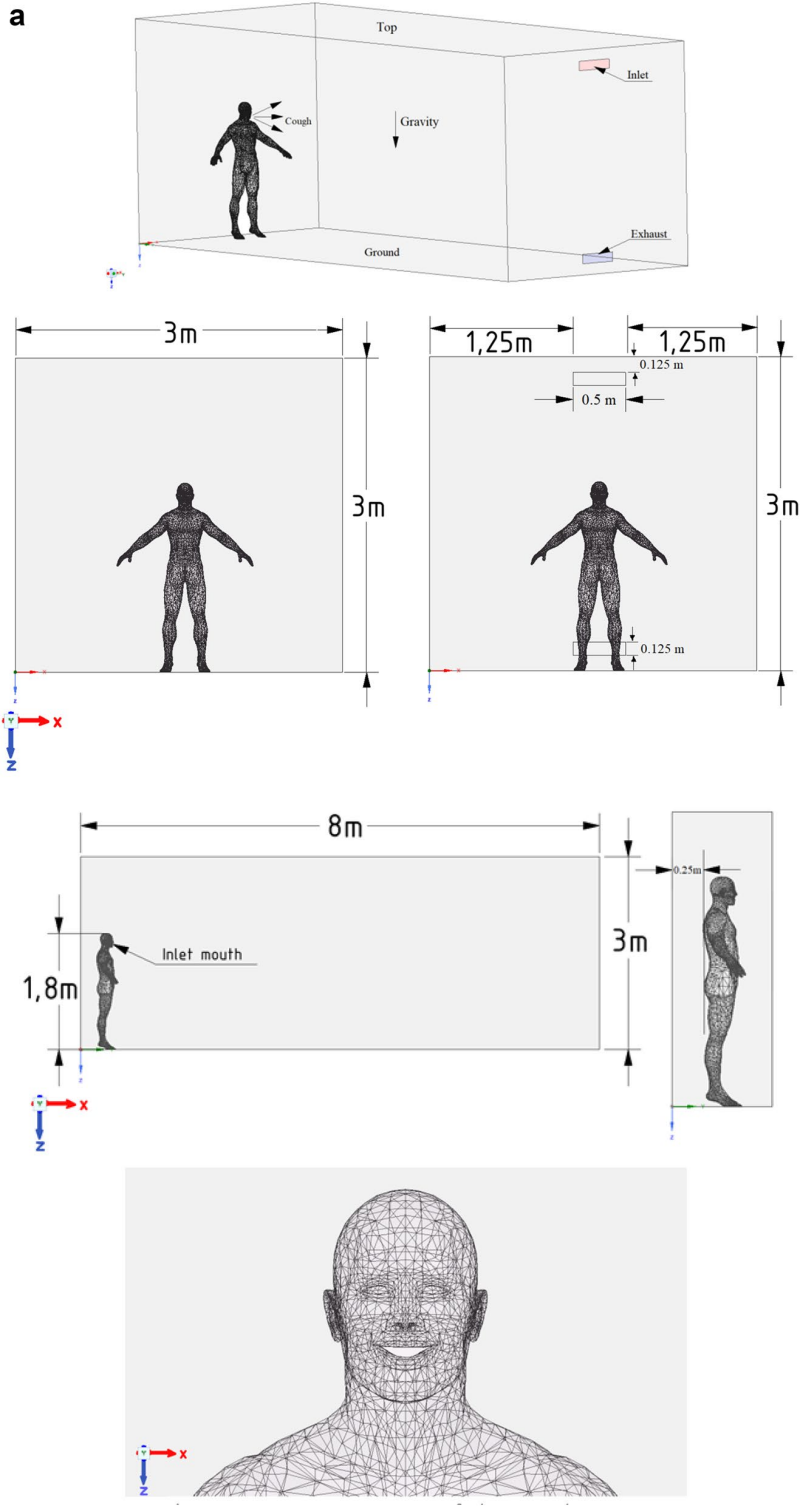

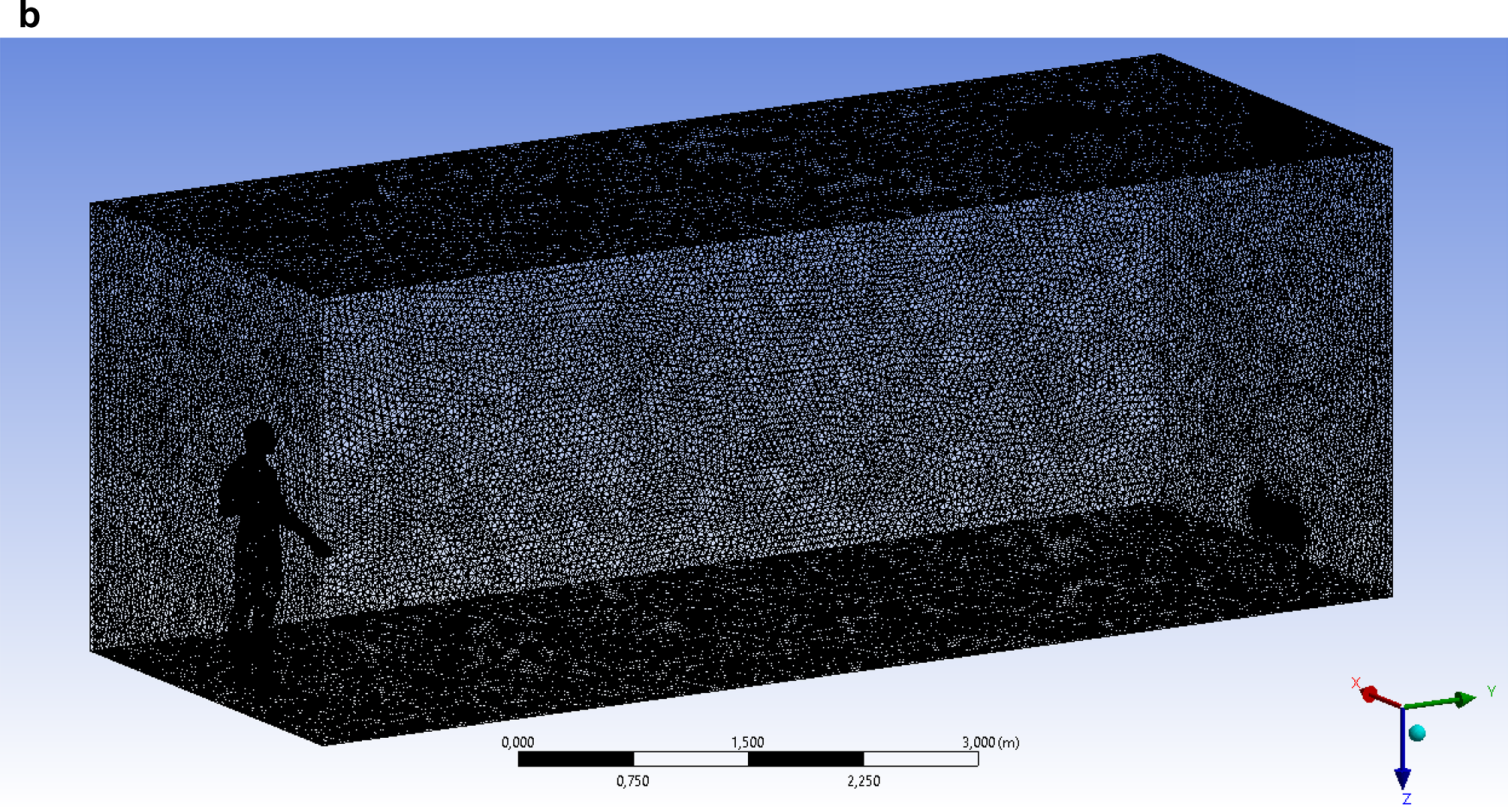
(**a**) Geometry of the study area. (**b**) Computational grid of the study area.

In this study, different velocities for the propagation of a particle when coughing in a room were examined. For this study, a full-scale room with dimensions X × Y × Z = 8 × 3 × 3 m was taken, which is used as an internal environment for the current simulation. The height of a person inside the room is 1.8 m and, accordingly, the height of the mouth of a polluting person from the floor is about 1.65 m. The full-scale room is ventilated with a single grate on the side wall, the grating size is 0.5 m × 0.125 m, and the same size hood is located at the bottom of the same wall. There is also one person in the room on the opposite side of the entrance and exit. A person’s cough (sneeze) indoors expels particles (respiratory droplets) of various sizes (Fig. 5a). Several scenarios were simulated to fully investigate the effects of different particle sizes, particle velocities and ventilation effects.

The rate of the normal breathing process is periodic, and the rate of the coughing or sneezing process is pulse. The rate of particles ejection from the mouth varies from 1 to 20 m/s, which is typical for various flows of breathing, coughing and sneezing, as reported in experimental data^26,31–33^. It should be noted that many people sneeze more than once per sneeze cycle. However, for this problem, a more simplified version of sneezing was considered, only once. In fact, the results below show that just a single sneeze or cough can cause long distance transport and a high concentration of ejected particles, which means that repeated sneezing or coughing, can cause a higher concentration. For a complete study, 9 scenarios were modeled, presented in Table 1.

**Table 1:** Simulation scenario.

Scenario	Particle ejection velocity (m/s)	Supply air velocity [m/s]	Particle diameter [m]
1	*V* = 1	0	1 × 10^–6^ to 1 × 10^–4^
2	*V* = 6	0	1 × 10^–6^ to 1 × 10^–4^
3	*V* = 20	0	1 × 10^–6^ to 1 × 10^–4^
4	*V* = 1	0.5 (0.038 kg/s)	1 × 10^–6^ to 1 × 10^–4^
5	*V* = 6	0.5 (0.038 kg/s)	1 × 10^–6^ to 1 × 10^–4^
6	*V* = 20	0.5(0.038 kg/s)	1 × 10^–6^ to 1 × 10^–4^
7	*V* = 1	1 (0.077 kg/s)	1 × 10^–6^ to 1 × 10^–4^
8	*V* = 6	1 (0.077 kg/s)	1 × 10^–6^ to 1 × 10^–4^
9	*V* = 20	1 (0.077 kg/s)	1 × 10^–6^ to 1 × 10^–4^

Unlike normal breathing, sneezing or coughing can form a large number of droplets with a diameter of about 10^–4^ to 10^–3^ m. However, they will split into smaller particles 10^–6^ to 10^–4^ m in size in a very short time. Therefore, the particle diameters in the simulation were in the range of 10^–6^ to 10^–4^ m. Figure 5b shows a three-dimensional (3D) computational mesh of the studied area, the total number is more than 5,093,129 elements. Since the accuracy of the results is highly dependent on the size of the computational grid, condensation around the mouth and body is used. This method helps to optimize computational costs by reducing the total number of cells for a given complex geometry. For the simulation, the optimal computational grid was chosen. And with a decrease in the number of the computational grid elements, it did not greatly affect the obtained numerical solutions. Ejection of particles from behind the mouth is realized from 0.1 to 0.3 s. An experiment was carried out in Busco et al., where sneezing was induced by stimulation of the nasal mucosa of a healthy adult male. During the sneezing experiments, only one person was allowed to enter the room.

The results of this study showed that the total duration of one sneeze was approximately 0.1925s. Therefore, in this work, the rate of emission of polluting particles from the mouth is given by the following formula

$$\left\{ {\begin{array}{*{20}l} {u = V,} \hfill & {10.1 \le t \le 10.25} \hfill \\ {u = V\sin (2\pi t),} \hfill & {10.25 < t \le 10.5} \hfill \\ {u = \sin (2\pi t),} \hfill & {else} \hfill \\ \end{array} } \right..$$

The particle density is assumed to be 600 kg/m^3^, which is the approximate density of particles or droplets of a mixture of water and air. In fact, since the particles are small, the particle density does not significantly affect the transport and distribution of particles. The rest of the boundary conditions on the walls were specified as a wall, where all velocity components were equal to zero (U_i_ = 0).

**Figure 6:**
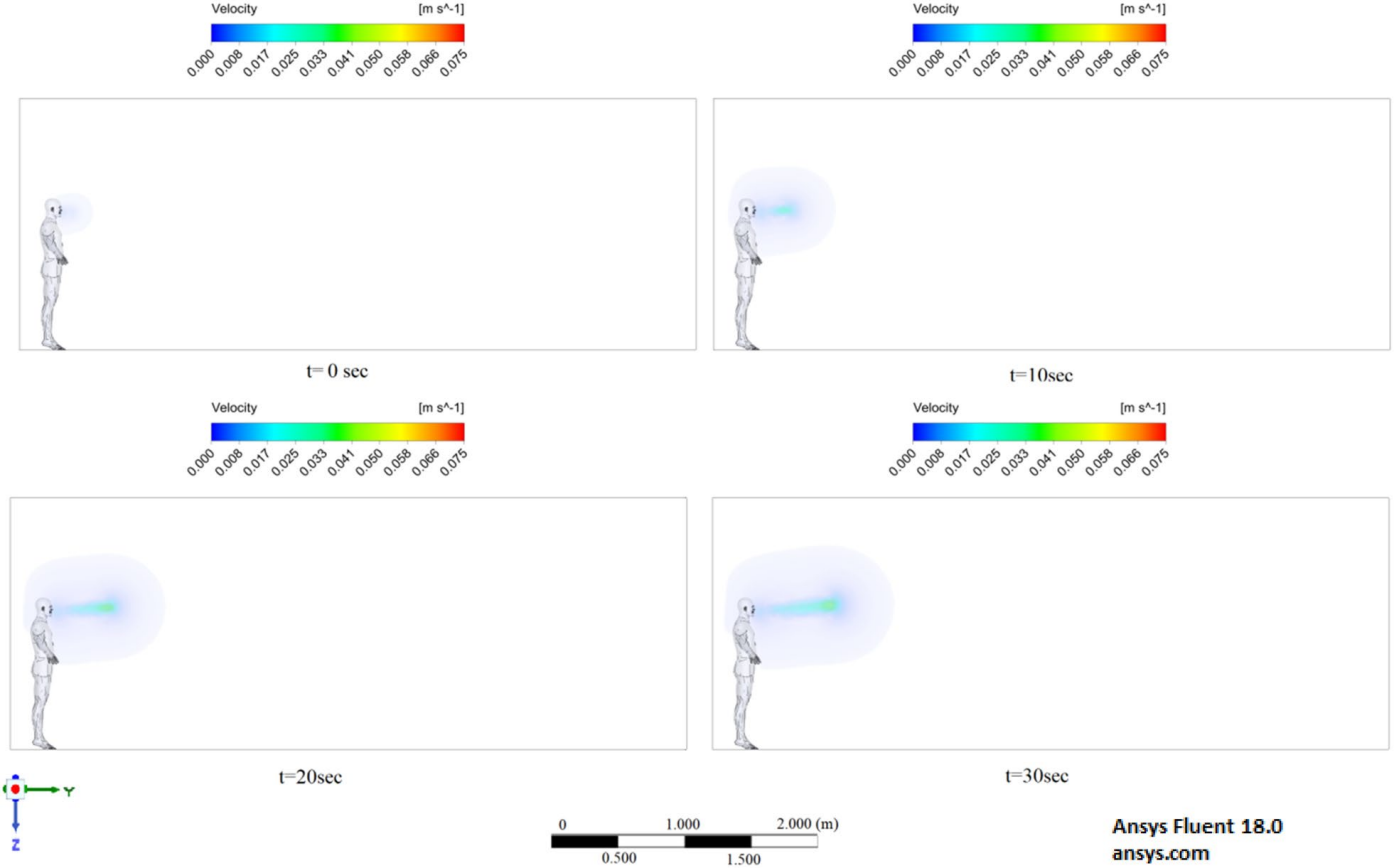
Cough = 1 m/s without ventilation.

**Figure 7:**
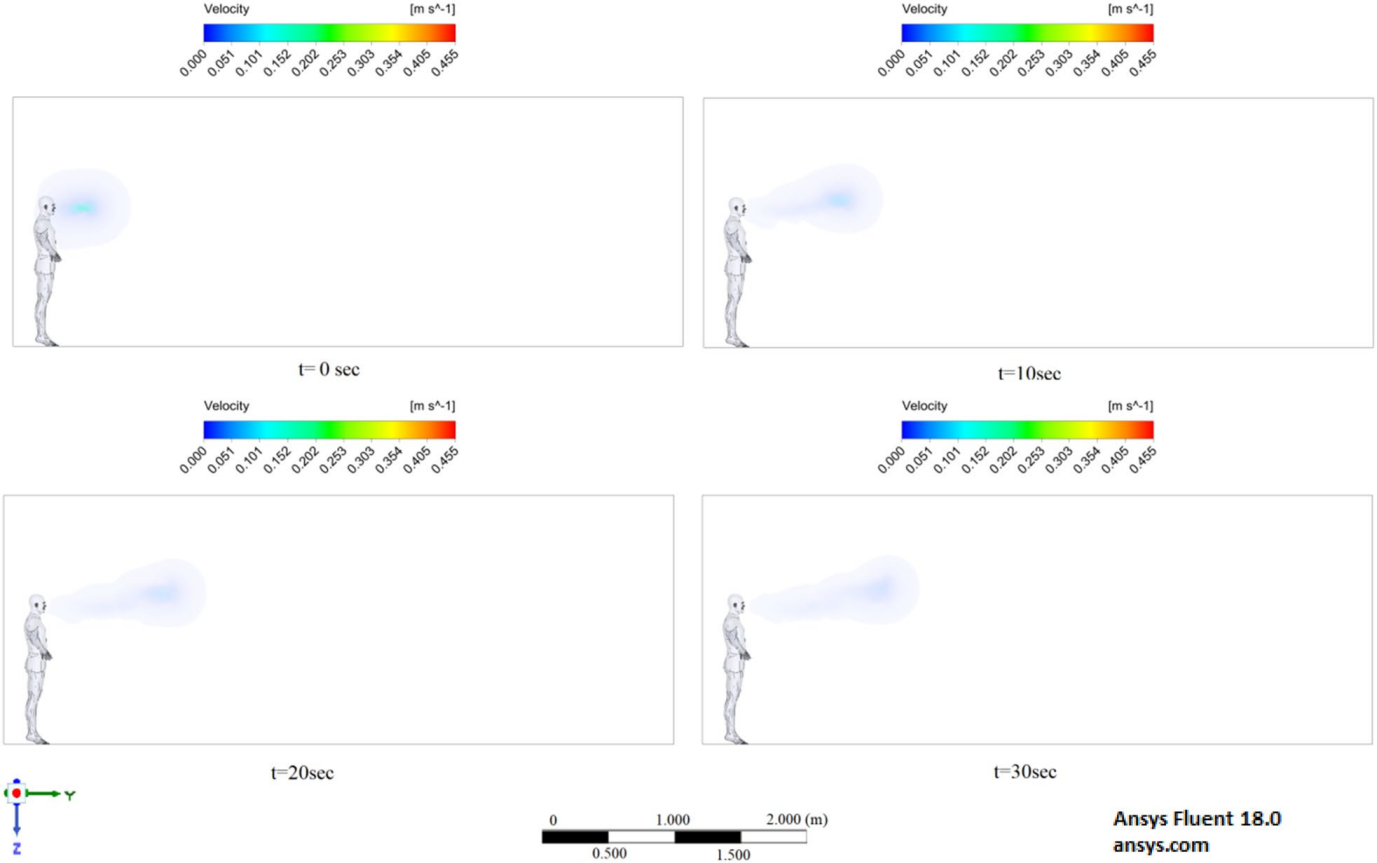
Velocity contours for cough = 6 m/s without ventilation.

**Figure 8:**
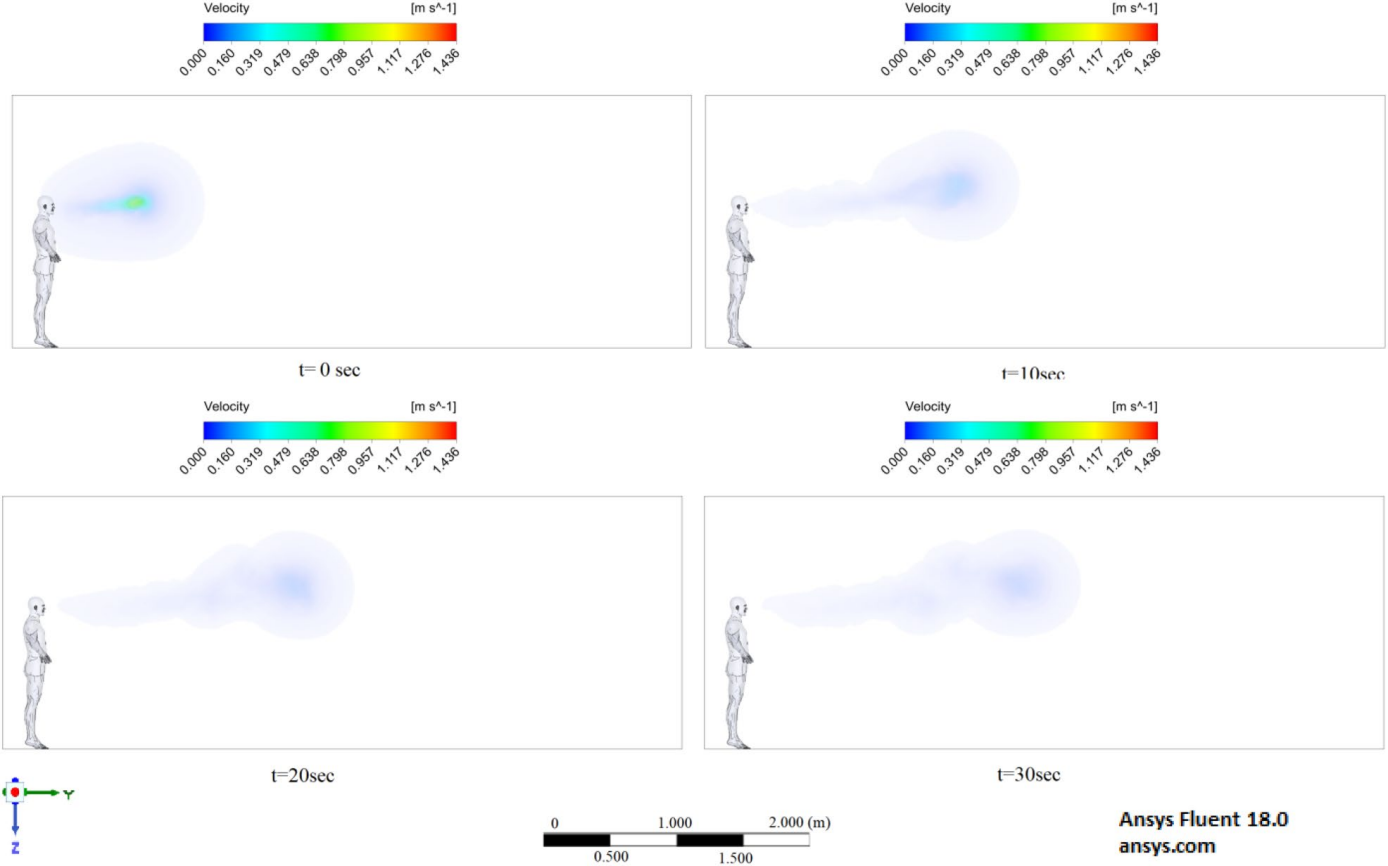
Velocity contours for cough = 20 m/s without ventilation.

**Figure 9:**
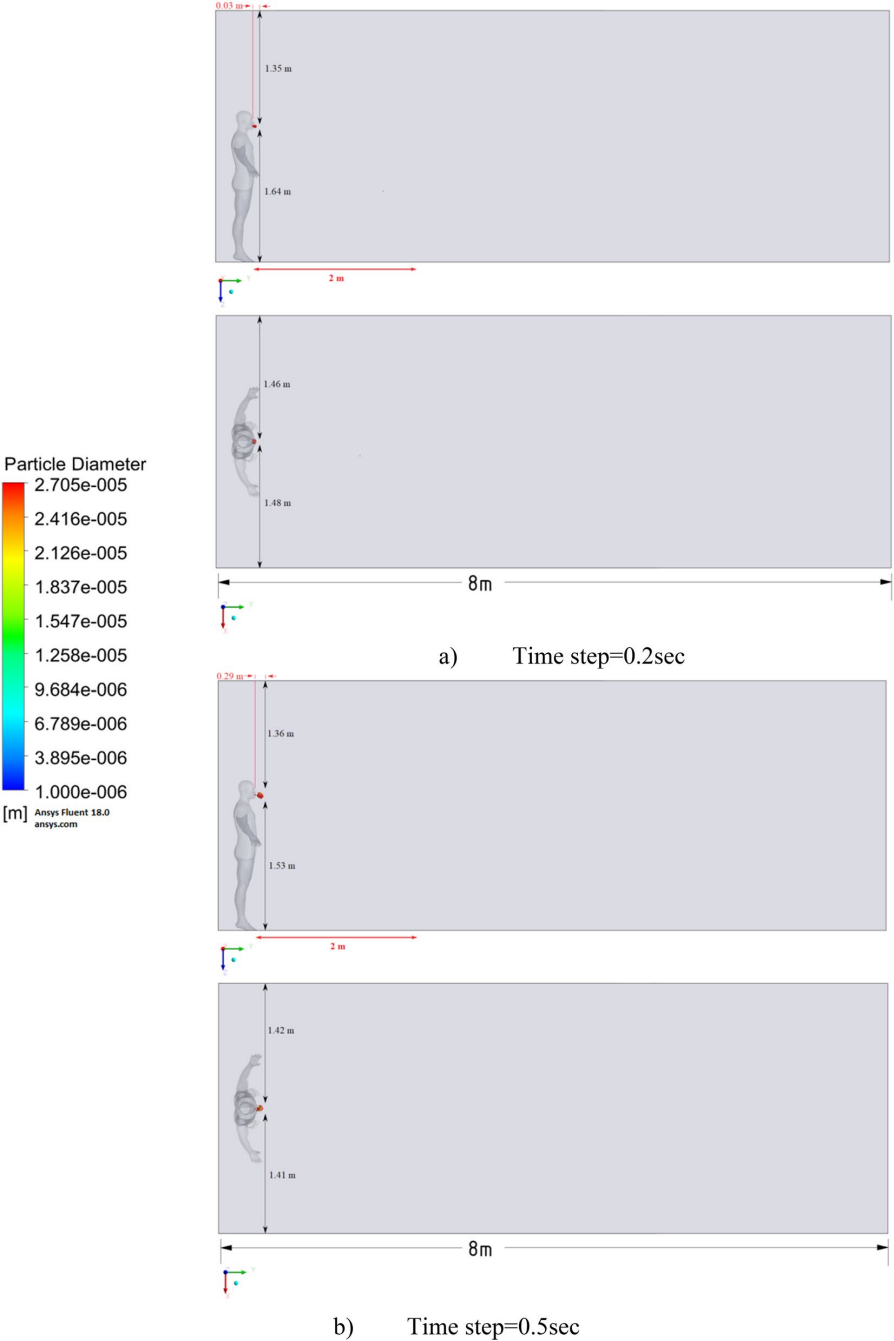

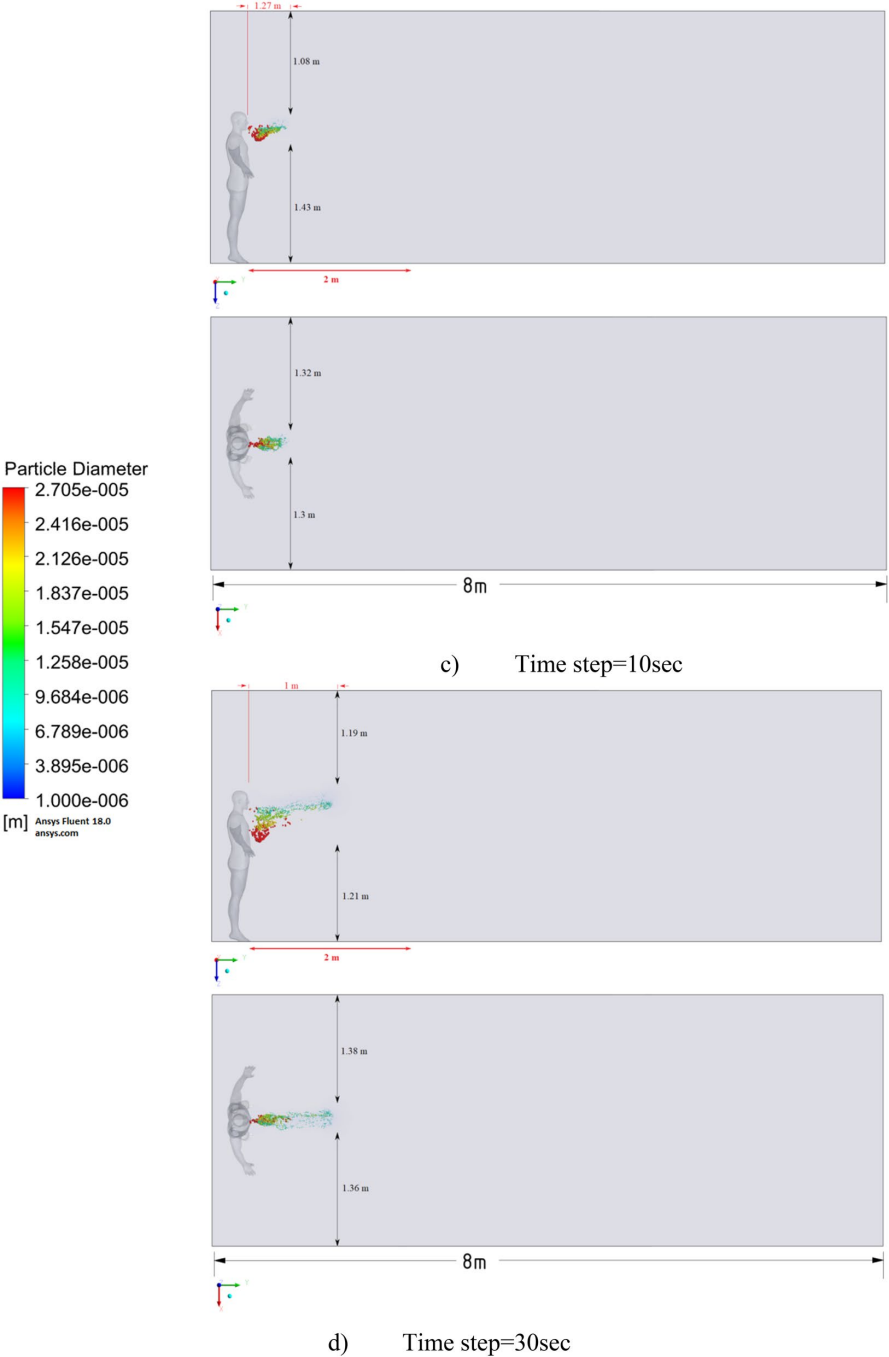
Distribution of the particles for cough = 1 m/s without ventilation.

**Figure 10:**
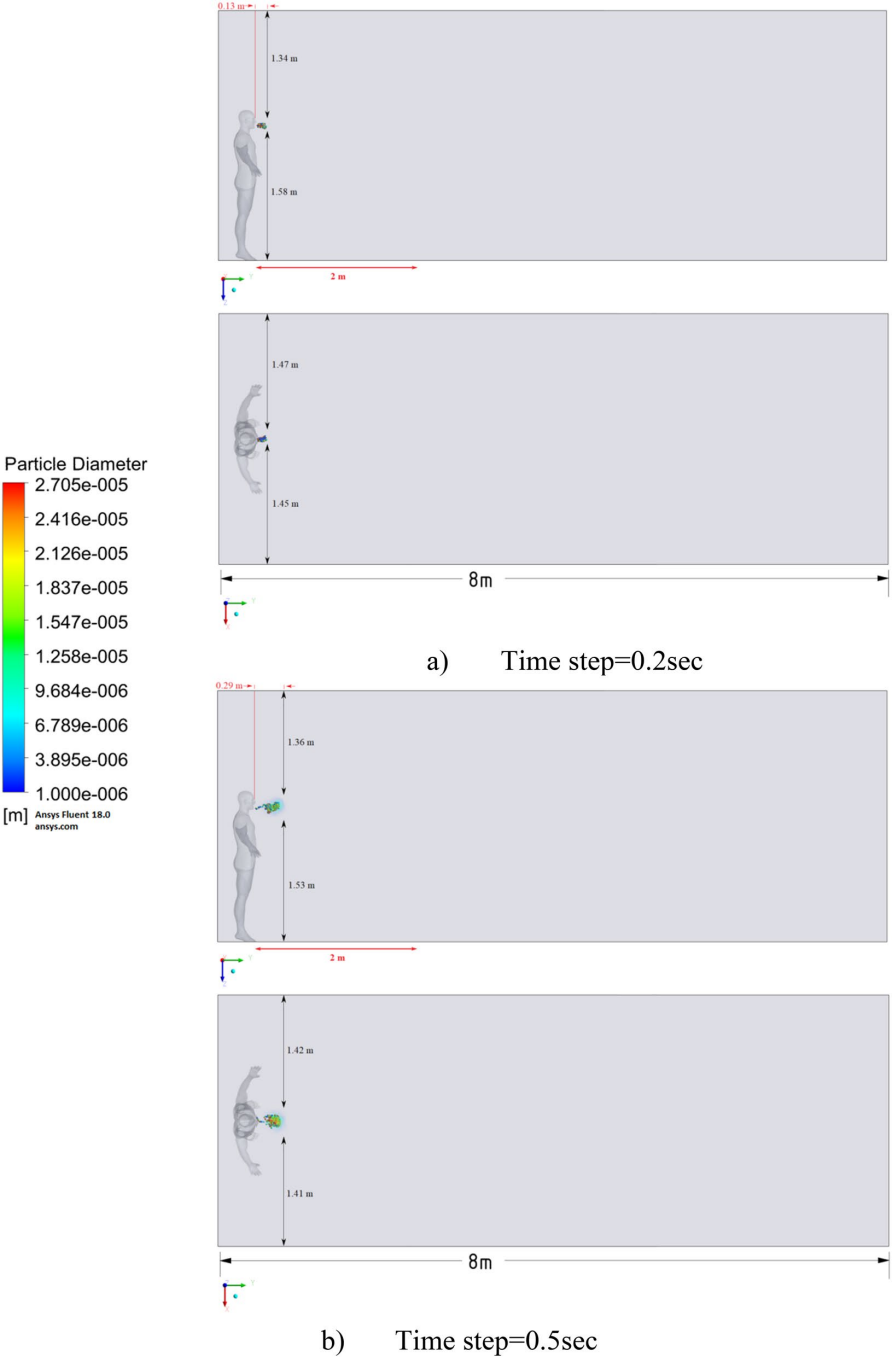

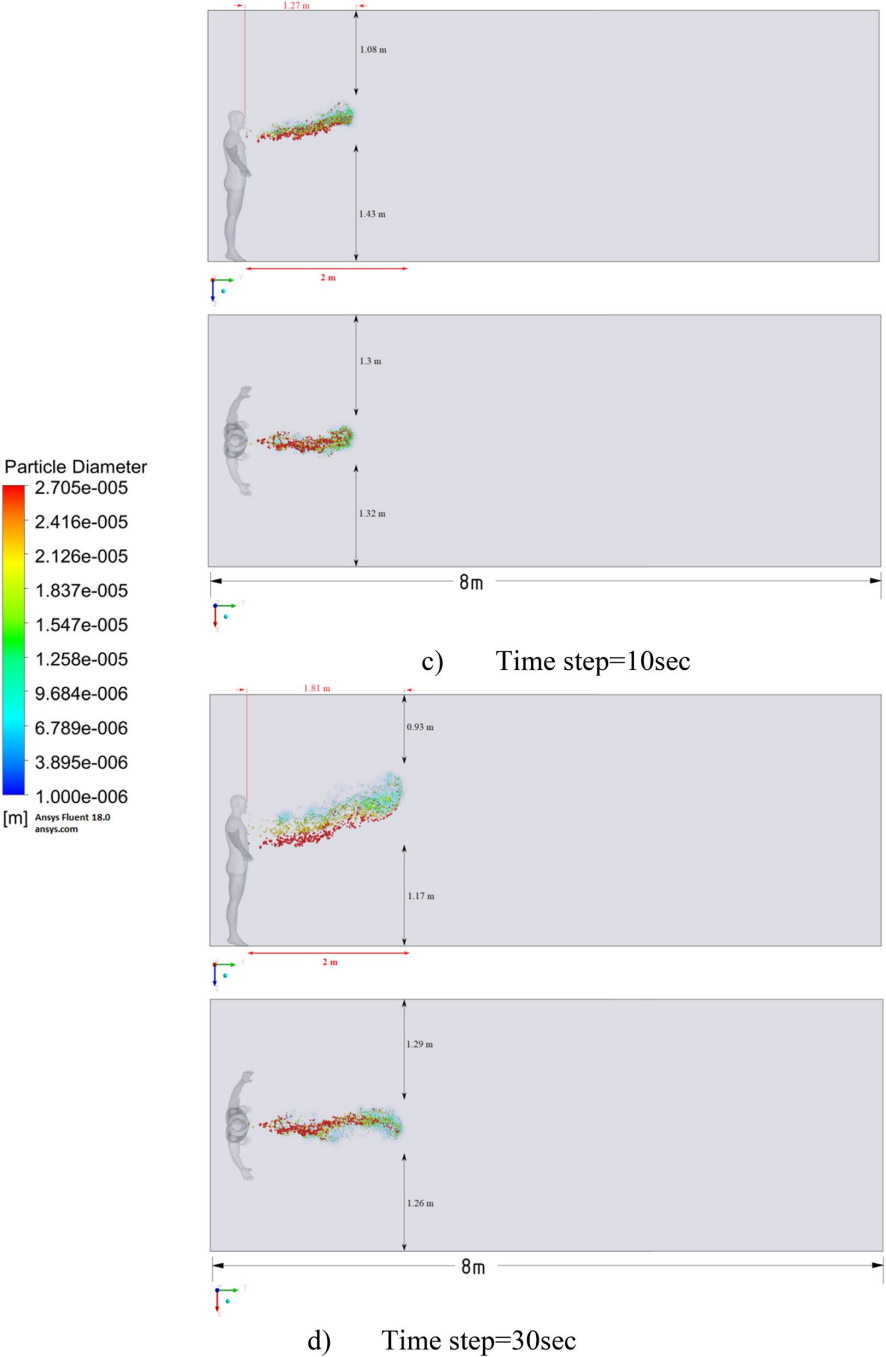
Distribution of the particles for cough = 6 m/s without ventilation.

**Figure 11:**
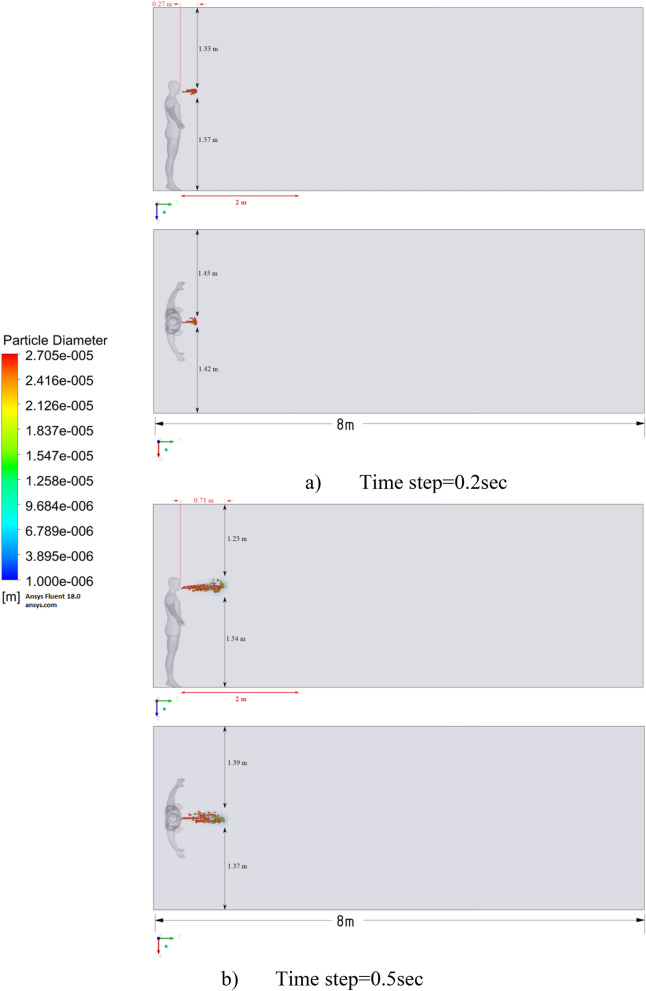

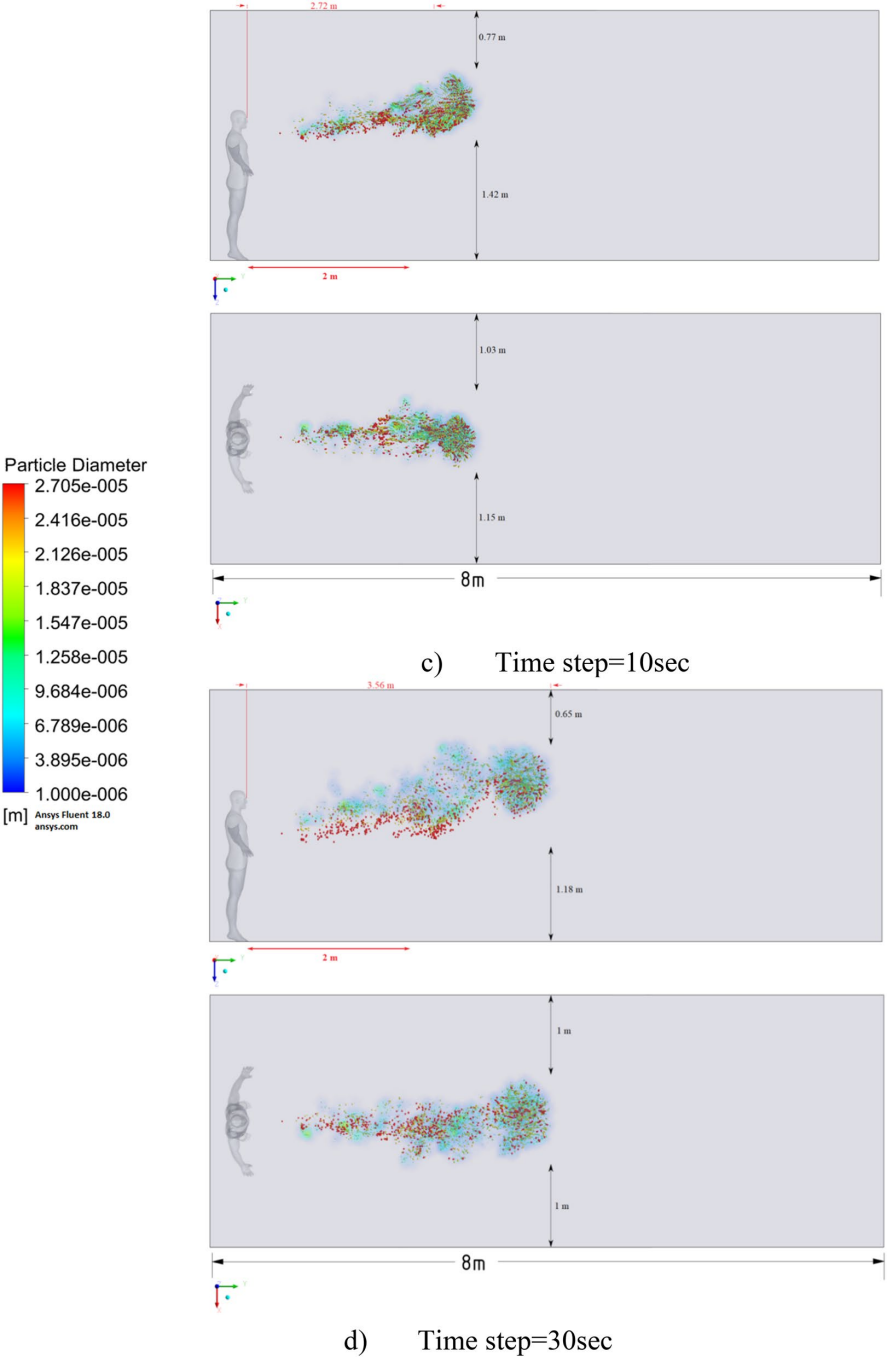
Distribution of the particles for cough = 20 m/s without ventilation.

Figures 6, 7 and 8 show the velocity results of scenarios 1–3 at different points in time. From the results presented in Figs. 6, 7 and 8, it can be seen that different velocity modes have a very strong effect on the flow distance in a closed room. As expected, an increase in velocity leads to an increase in flow. The results of Fig. 8 show that in 30 s the stream ejected from the mouth can cover a distance of 4 m. However, this does not mean that particles can also be transported by 4 m, since friction forces and gravity also act on the particles. The results of particle transport at different times are shown in Figs. 9, 10 and 11.

The results of Fig. 9 of Scenario 1 clearly show how particles with large diameters settle due to the force of gravity and due to the large area of the particle, since particles with larger sizes are more susceptible to drag forces. It is also worth noting that in scenario 1 in 30 s the particles cover a distance of 1 m in length and settle at 0.44 m in height, which does not exceed the social distance recommended by WHO (2 m)^2,3^.

Figure 10 shows the results of particle propagation at different points in time when coughing or sneezing 6 m/s. These results show that in 30 s, particles are transported 1.81 m in length and fall 0.48 m in height. It is also worth noting that the distribution of particles across the width of the room is symmetrical about the two side walls. The results of Figs. 9 and 10 show that in scenarios 1 and 2 the particles do not overcome the recommended social distance of 2 m.

**Figure 12:**
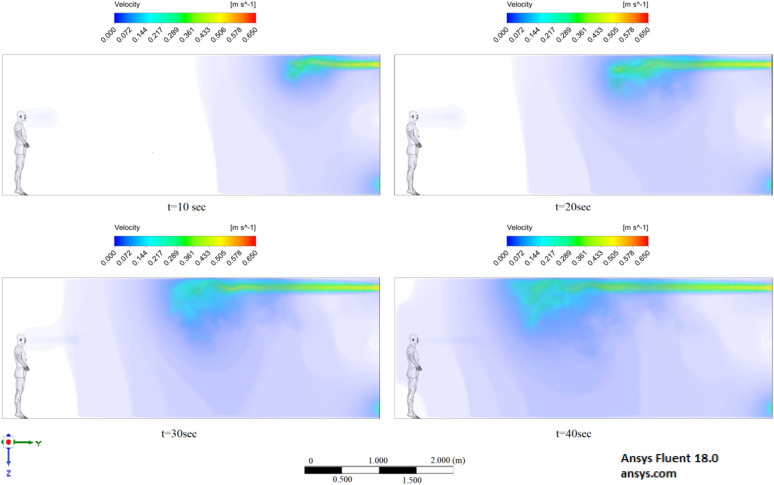
Velocity contours for cough = 1 m/s and ventilation = 0.5 m/s.

**Figure 13:**
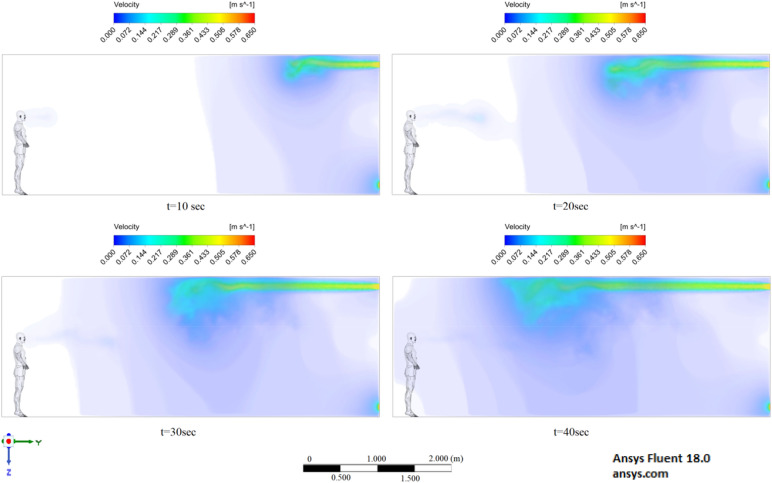
Velocity contours for cough = 6 m/s and ventilation = 0.5 m/s.

**Figure 14:**
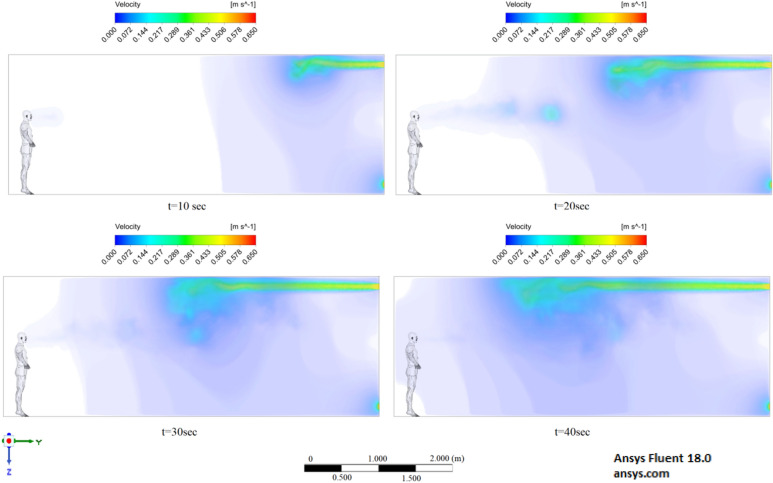
Velocity contours for cough = 20 m/s and ventilation = 0.5 m/s.

**Figure 15:**
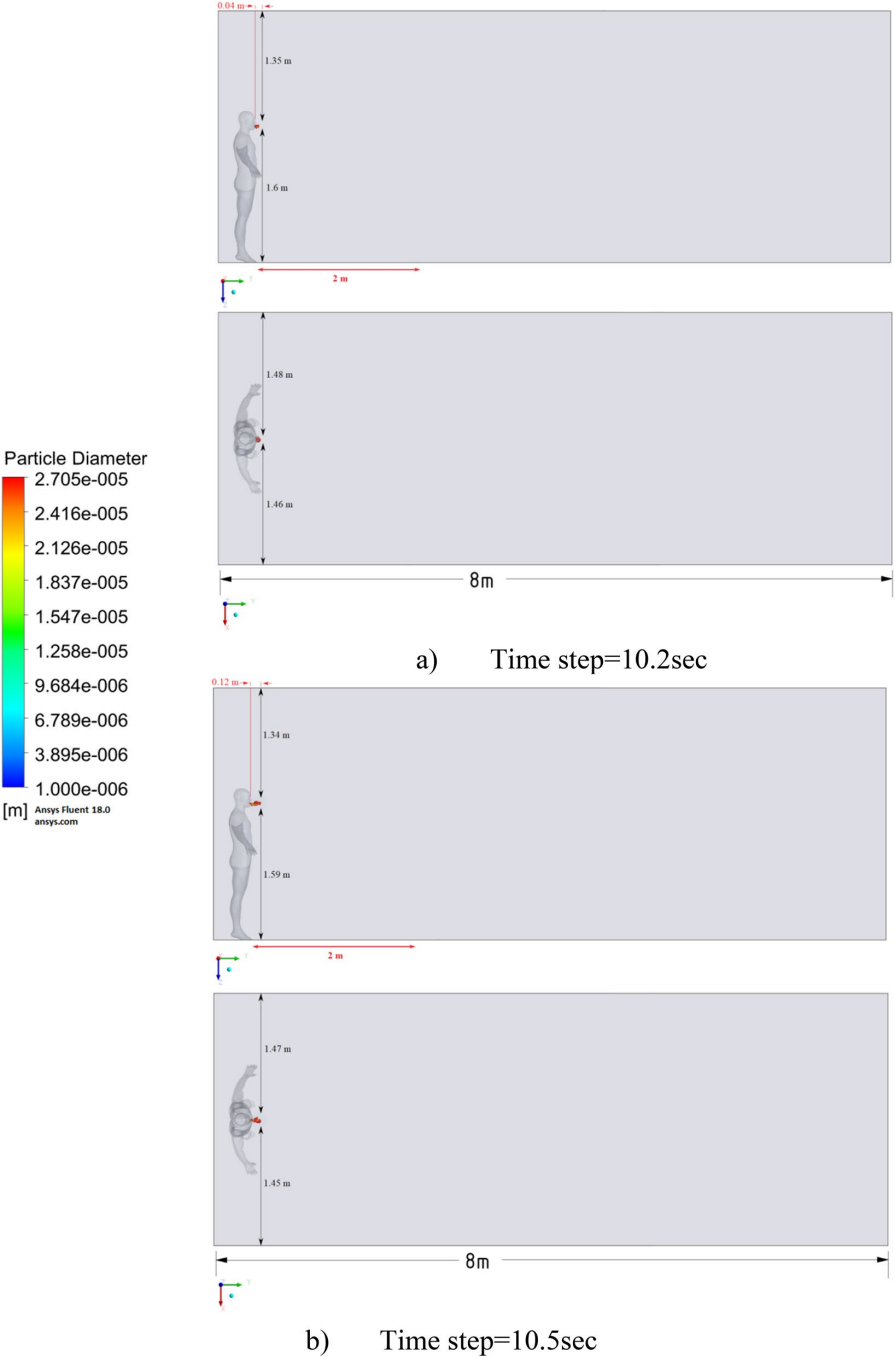

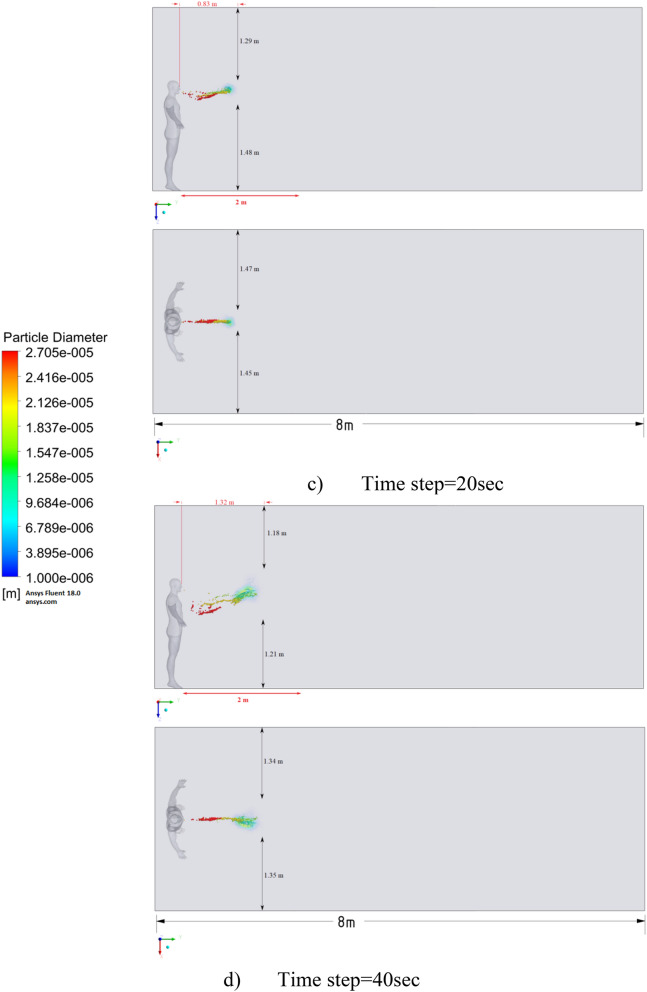
Distribution of the particles for cough = 1 m/s and ventilation = 0.5 m/s.

**Figure 16:**
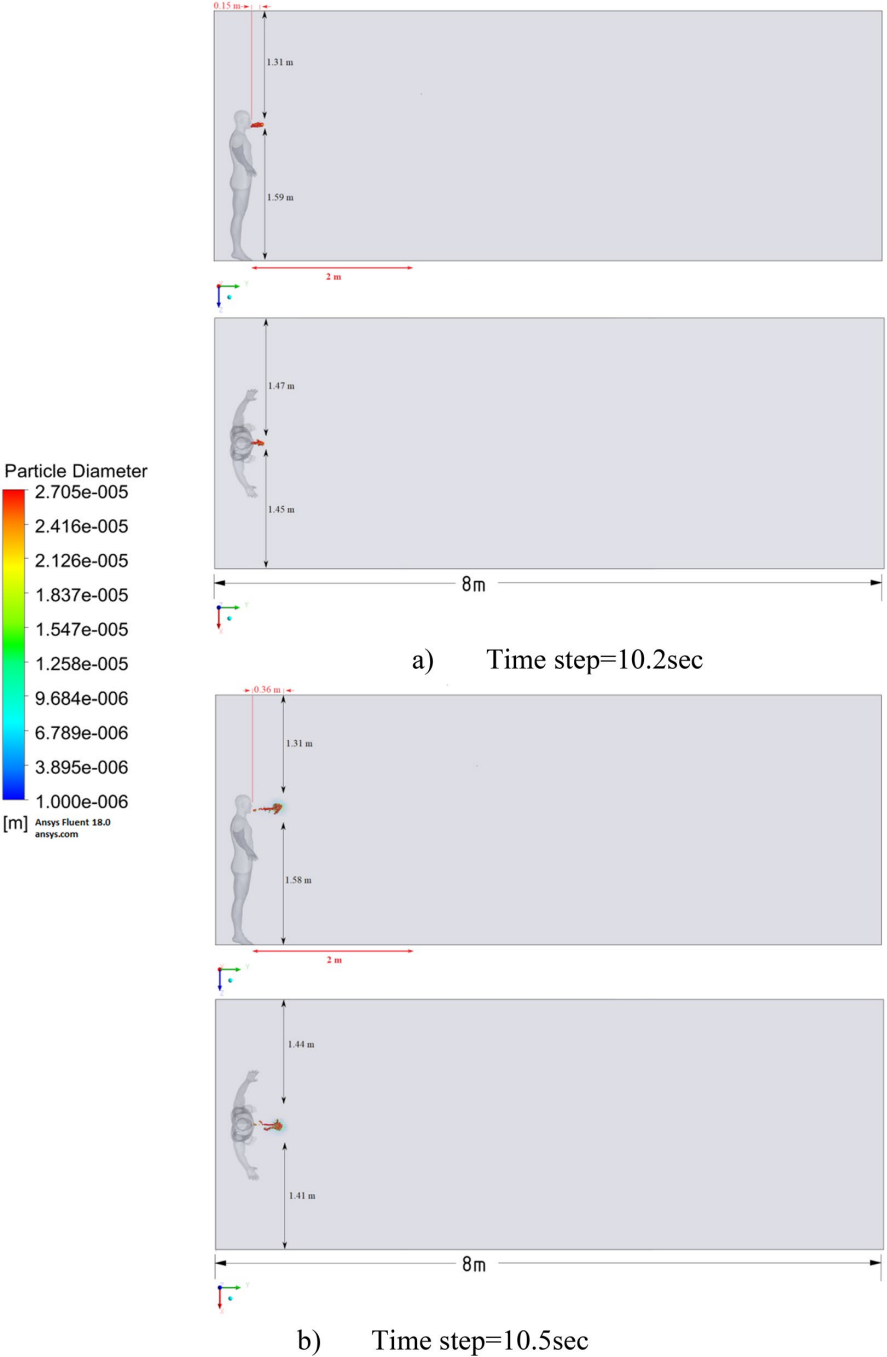

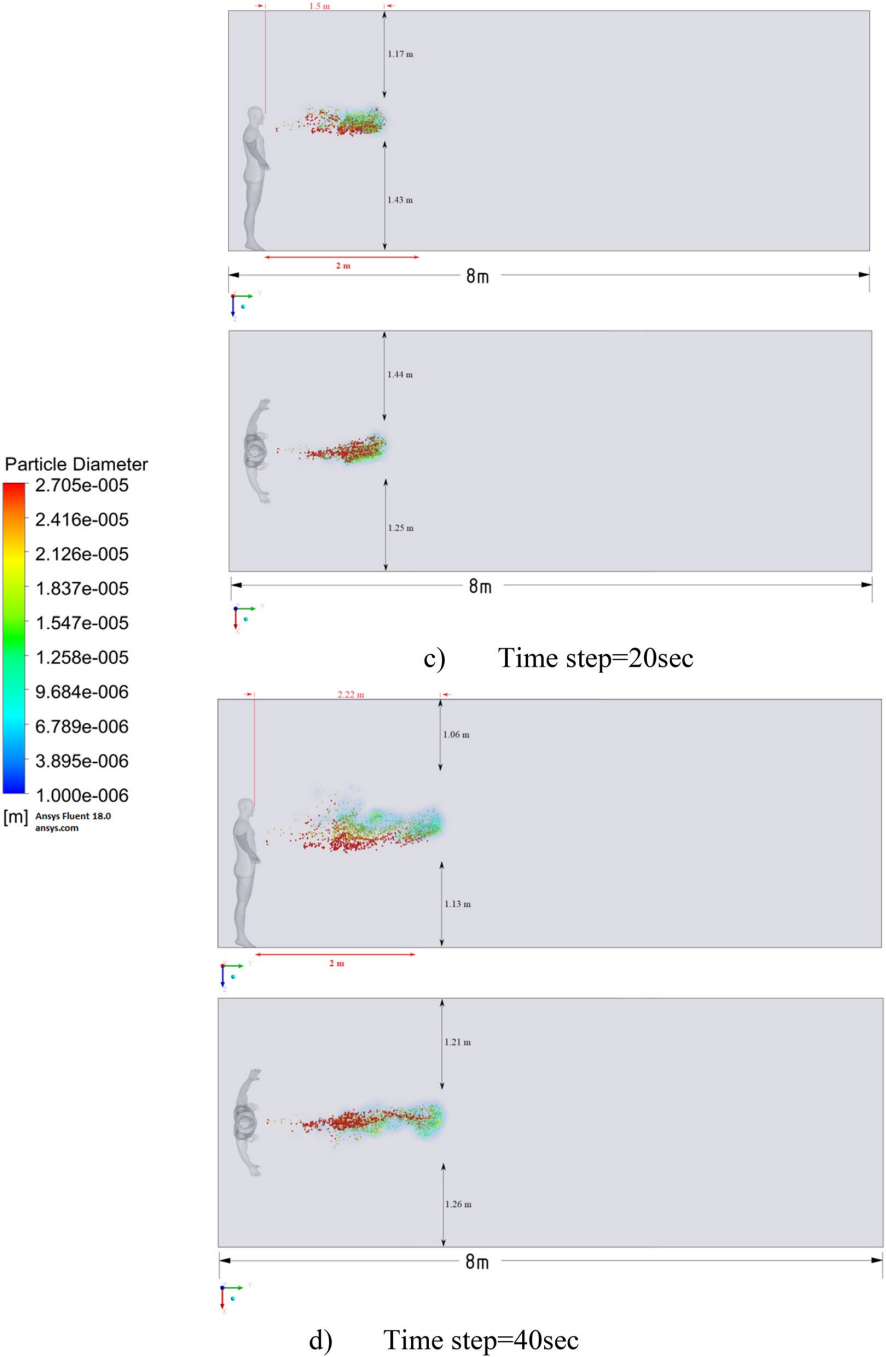
Distribution of the particles for cough = 6 m/s and ventilation = 0.5 m/s.

**Figure 17:**
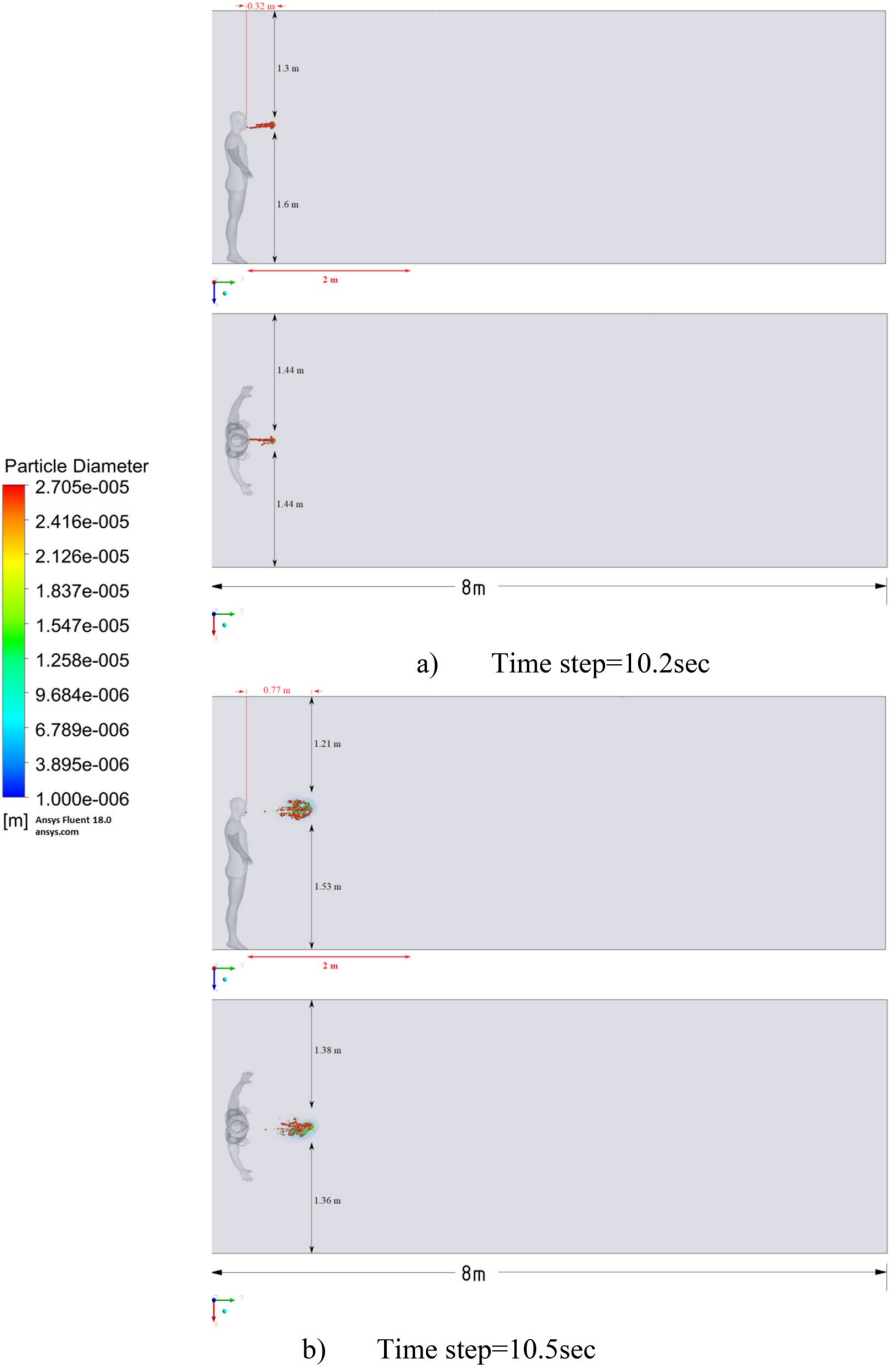

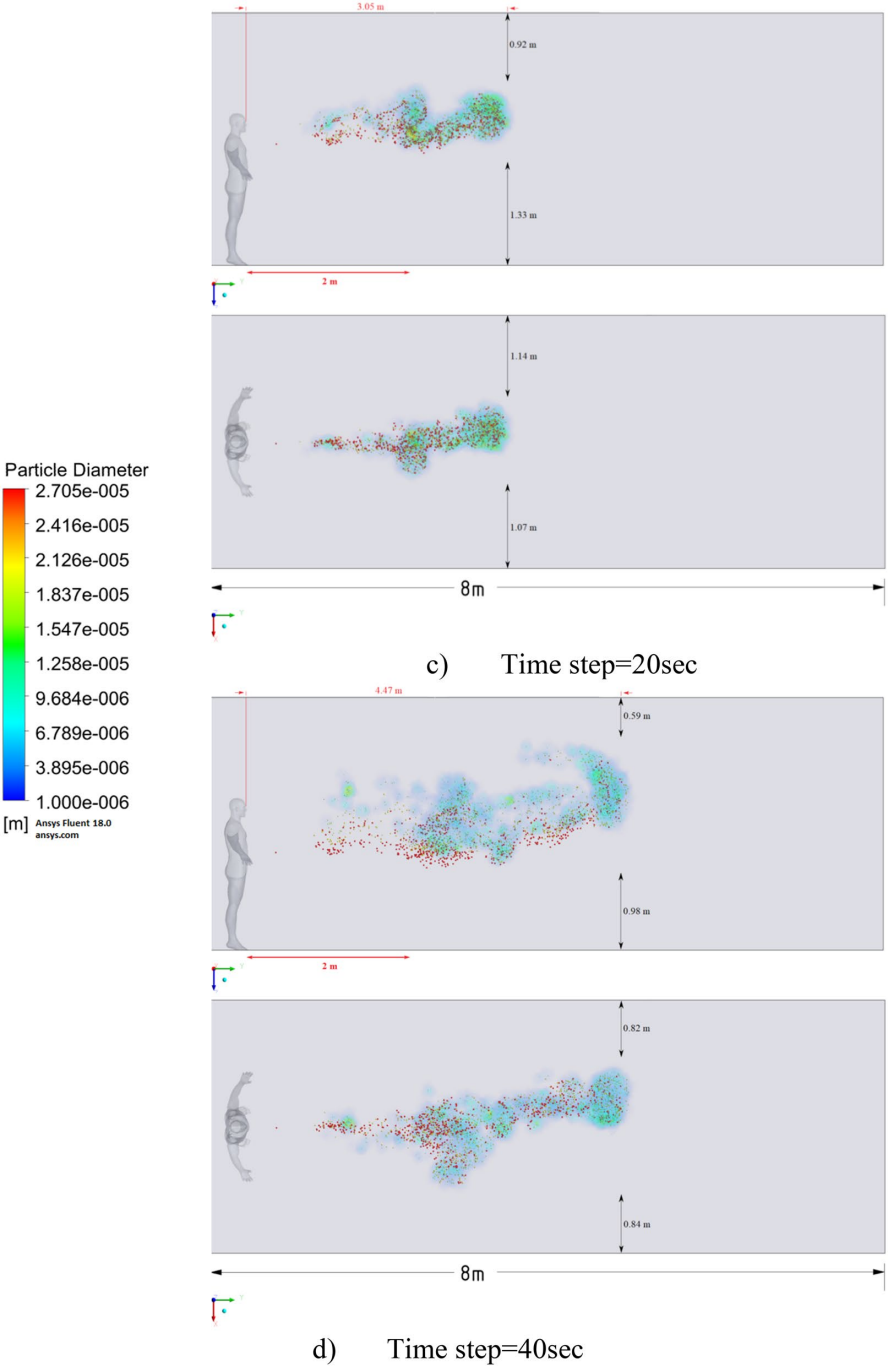
Distribution of the particles for cough = 20 m/s and ventilation = 0.5 m/s.

The presented results of scenario 3 in Fig. 11 clearly show that at 30 s the particles settle by 0.47 m in height and are transported by 3.56 m in length, exceeding the social distance recommended by the WHO. This means that in scenario 3 it is dangerous for people who are in this range. It is also worth noting a symmetric increase in the spread of particles in width and an increase in the height of dispersion of particles, where the distance from the side walls decreased to 1 m and the distance from the ceiling decreased to 0.65 m. Due to the relatively small volume of air flow during breathing, the process of inhalation and exhalation has little effect on the structure of the air flow in the room. Therefore, in order to avoid the free spread of contaminants and particles in confined spaces, ventilation schemes are designed. Scenarios 4–9 were simulated to investigate the effect of ventilation in the room on particle propagation. In order to obtain numerical results as close to reality as possible, the ventilation system will already be in operation for the first 10 s, and then, from 10.1 s, particles are emitted due to a cough or sneeze to 10.3 s, then a simple inhalation and exhalation of a person is realized without the release of particles with various sizes. The results of a numerical study of scenarios 4–6 are presented in Figs. 12, 13, 14, 15, 16 and 17.

Figures 12, 13 and 14 show the numerical results of the room flow rates at different times. In these results, it can be seen the ventilation flow which will affect the spread of particles. Ventilation at a speed of 0.5 m/s should have a positive effect on the diffusion and cleaning of air from polluting particles. Figures 15, 16 and 17 show the results of particle propagation in a room with ventilation of 0.5 m/s at different points in time. Comparing the results for scenarios 1 (Fig. 9) and 4 (Fig. 15), one can clearly see the effect of ventilation on particle transport. Ventilating the room at a speed of 0.5 m/s increases the spread of particles with different sizes, which increases the area of contamination. However, it must also be borne in mind that ventilation is designed to dissipate the concentration of pollutants in the room, and the ventilation flow should eventually clear the room by ventilation.

From the presented results in Fig. 16, it can be seen that in scenario 5, the particles overcome the recommended social distance in 40 s and are transferred to 2.22 m. Also, from the obtained numerical results, it can be seen that the particles spread along the width and height, increasing the contamination range. As in the results shown in Fig. 15, the ventilation of the room increases the area of contamination, but it should be noted that there is a dilution of the concentration of particles with air in the room, which leads to a decrease in the concentration of particles by 1 m^3^. So this dilution can play a positive role in the future, since diluted particles, falling on a person who stands on the other side of the room, can have little effect on him, which leads to a decrease in the risk of contracting an infectious disease.

**Figure 18:**
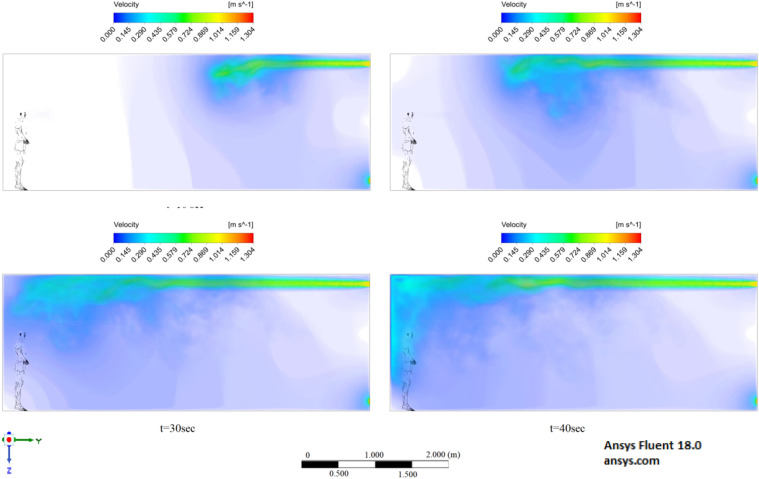
Velocity contours for cough = 1 m/s and ventilation = 1 m/s.

**Figure 19:**
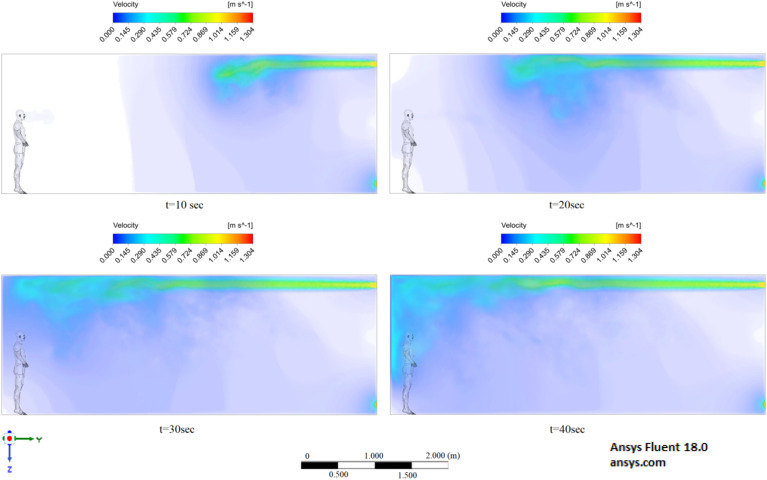
Velocity contours for cough = 6 m/s and ventilation = 1 m/s.

**Figure 20:**
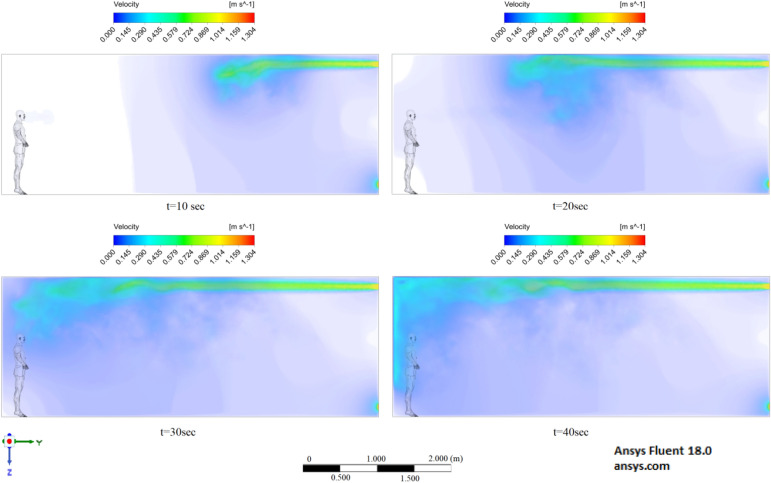
Velocity contours for cough = 20 m/s and ventilation = 1 m/s.

**Figure 21:**
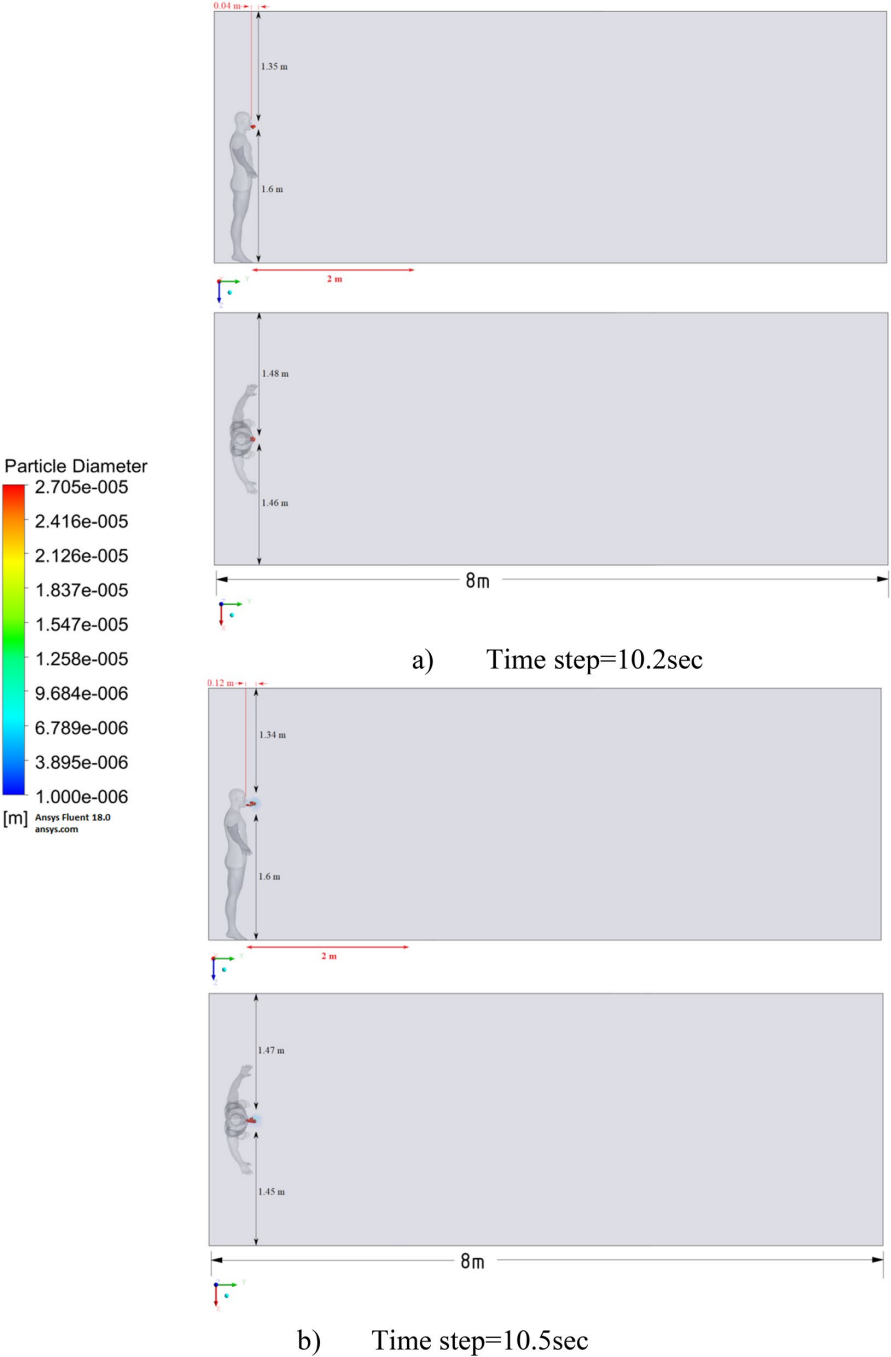

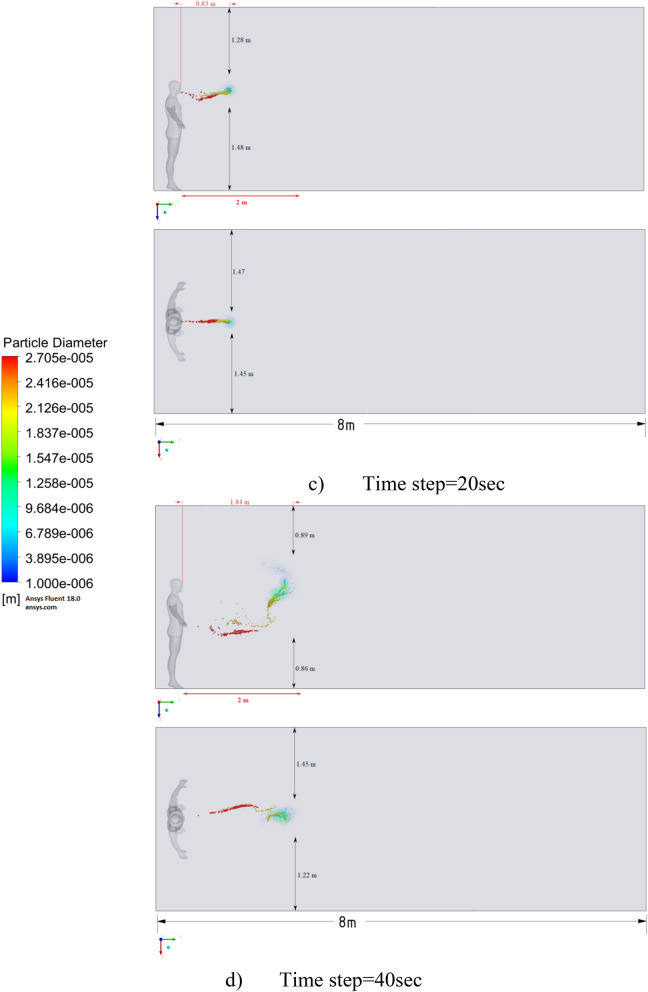
Distribution of the particles for cough = 1 m/s and ventilation = 1 m/s.

**Figure 22:**
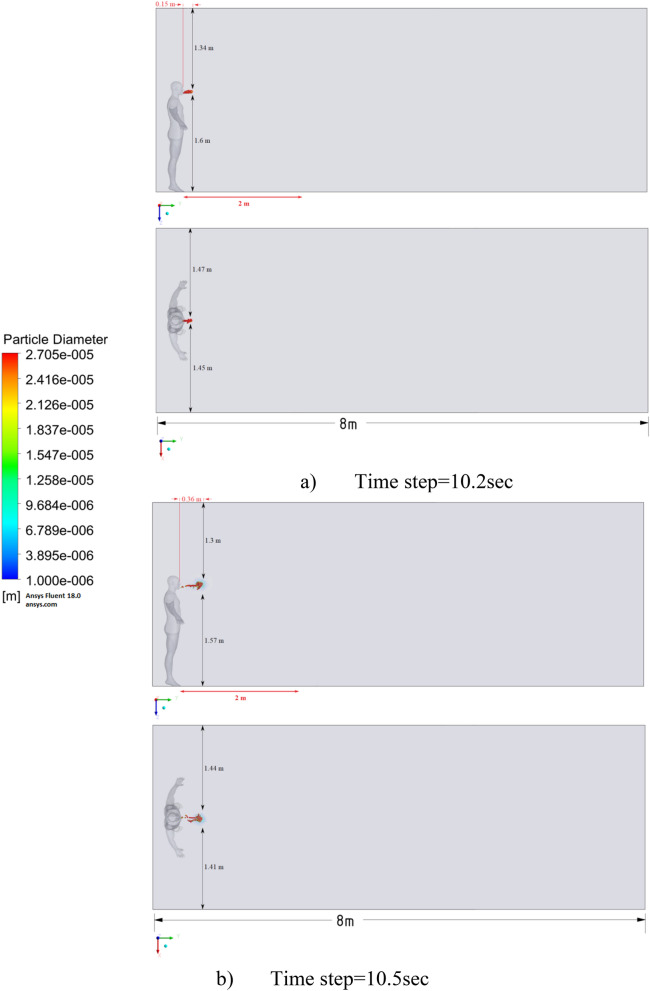

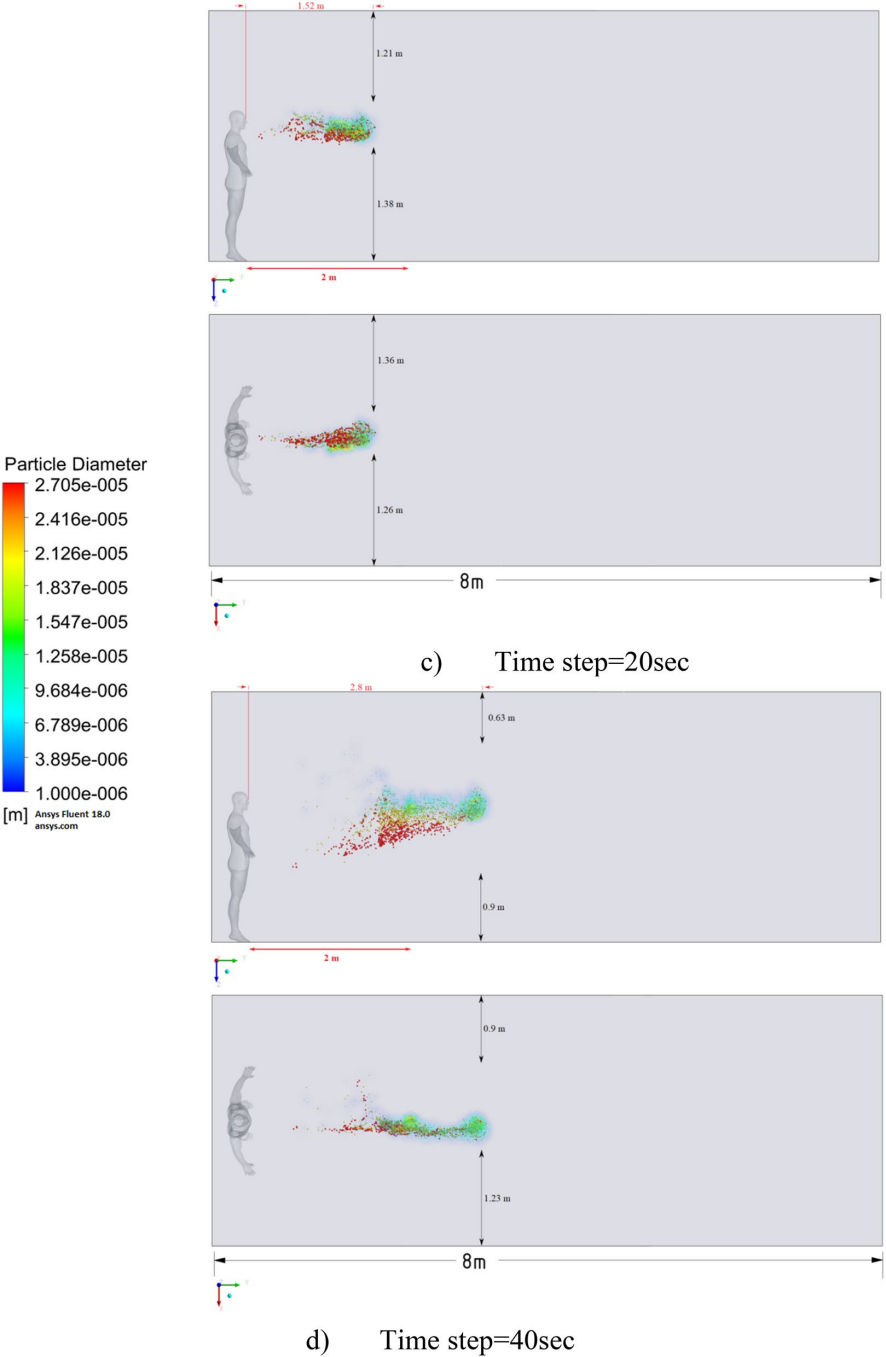
Distribution of the particles for cough = 6 m/s and ventilation = 1 m/s.

**Figure 23:**
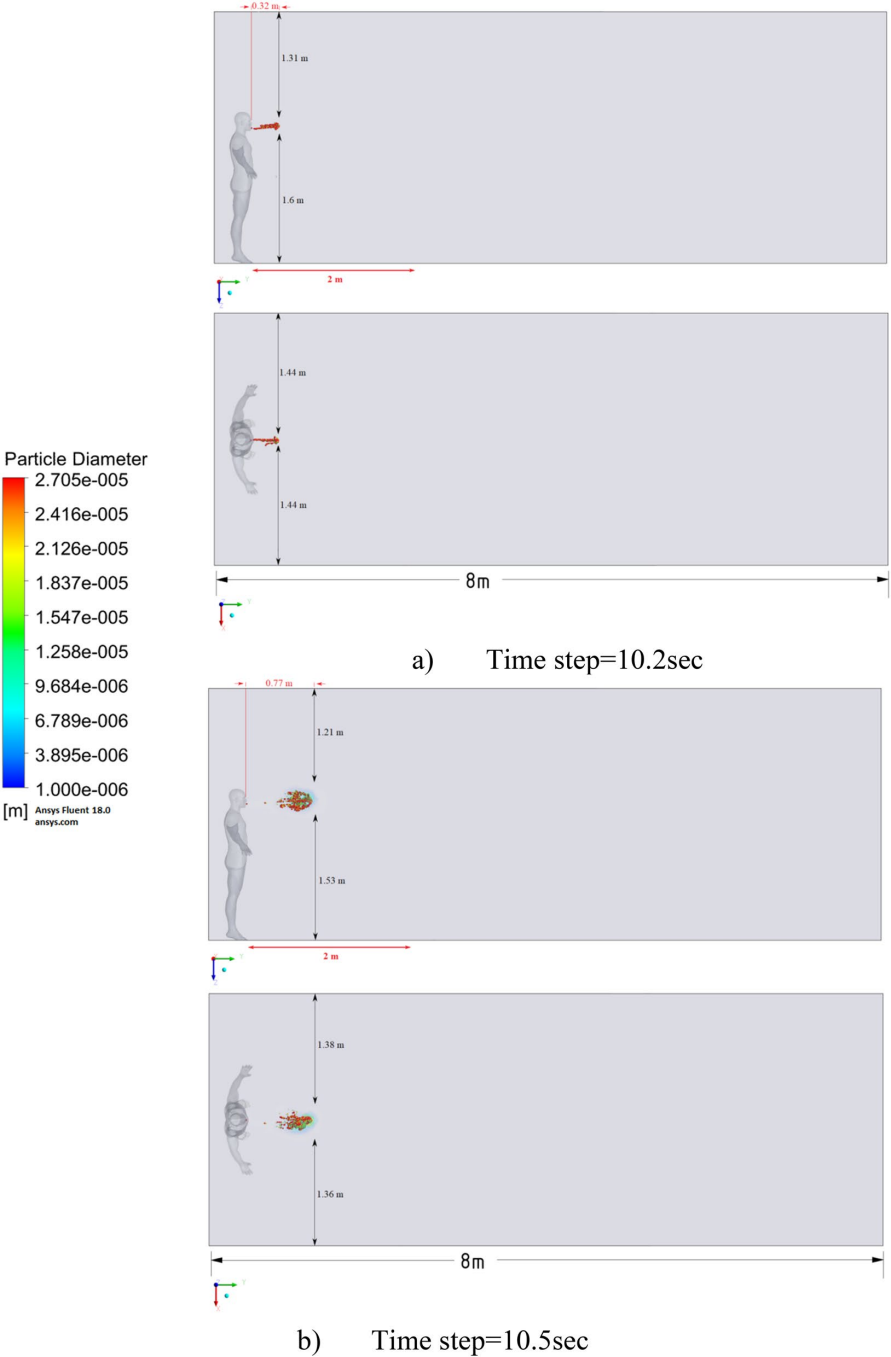

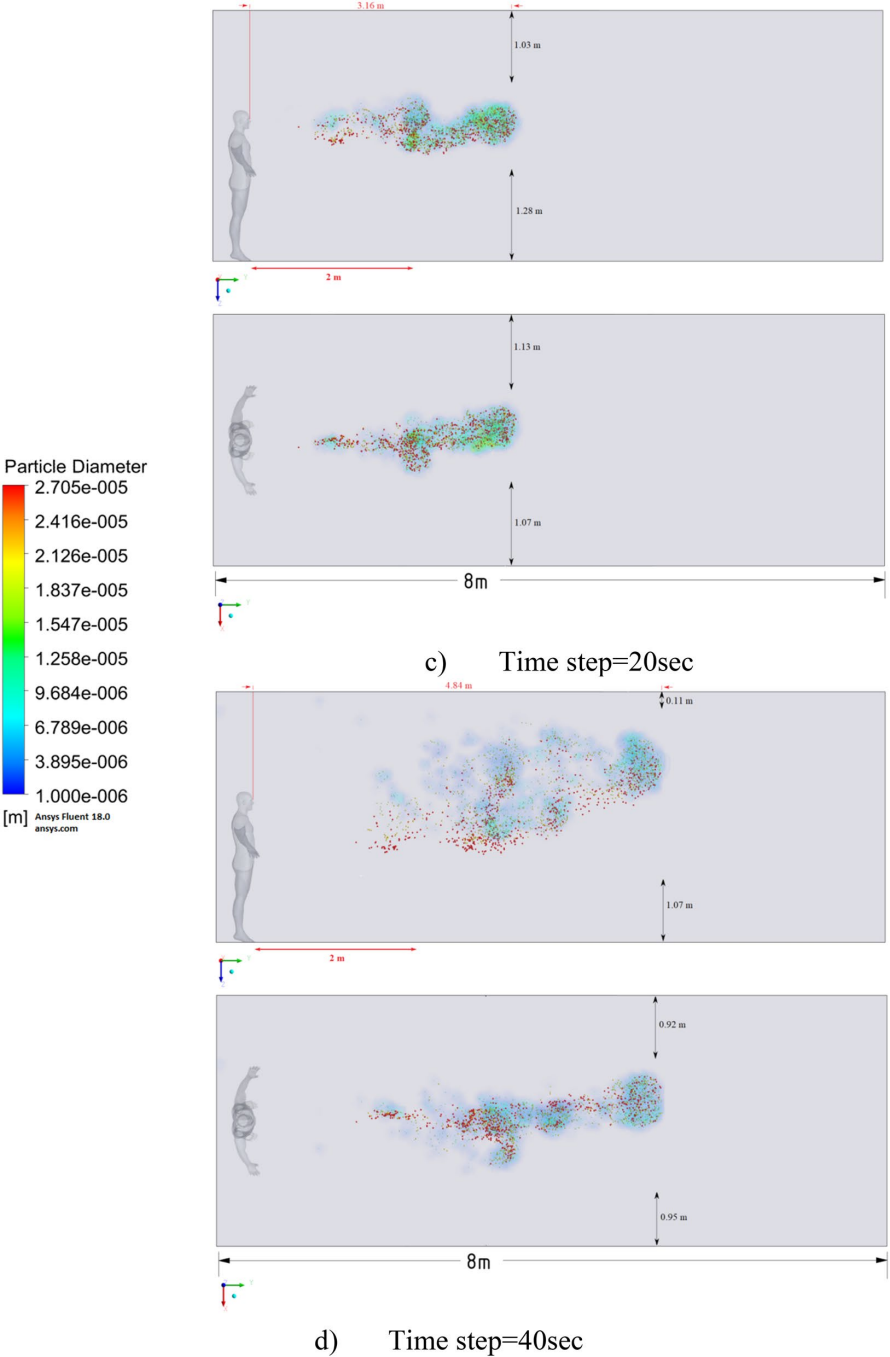
Distribution of the particles for cough = 20 m/s and ventilation = 1 m/s.

The results displayed in Fig. 17 illustrate the propagation of particles at different points in time. From these results, one can clearly see that in 40 s, pollutants are transported 4.47 m in length and 0.67 m in height. The range of propagation in all directions also increases. The results of Figs. 15, 16 and 17 clearly show the effect of room ventilation on the spread of particles with different sizes. Over time, a cloud of large particles settles gradually due to the force of gravity. For a complete study of the dependence on the room ventilation rate, scenario 7–9 was simulated. The results of scenarios 7–9 are presented in Figs. 18, 19, 20, 21, 22 and 23, where ventilation at a velocity of 1 m/s was considered.

From the results shown in Figs. 18, 19 and 20, it can be seen that the flow rates of a room with ventilation of 1 m/s are much higher, which can have a good effect on cleaning this room. Figures 21, 22 and 23 show the numerical results of particle propagation in a room with 1 m/s ventilation.

Figure 21 shows the numerical results of particle transport at different points in time for scenario 7. Under conditions of ventilation of 1 m/s and particle ejection at a velocity of 1 m/s, particles in 40 s are transferred 1.84 m in length and 0.79 m in height. From these results, it is worth highlighting that at 40 s in height, it can be seen the separation of particles by diameter, that is, particles with larger diameters are more susceptible to gravity, while particles with smaller diameters are more susceptible to momentum.

The presented results in Fig. 22 for scenario 8 also show an increase in the area of propagation of particles and settling of particles with large diameters. Under these conditions, particles are transported in 40 s 2.8 m in length and 0.75 m in height, which also exceeds the social distance.

Figure 23 shows the results of scenario 9, where it can be seen the maximum transport of particles around the room. In 40 s, the particles covered a distance of 4.84 m in length, and particles with large diameters also settled by 0.58 m in height. It is also worth noting that the particles with small diameters have almost reached the ceiling. The results of a numerical study of the propagation of particles showed that in 40 s, particles can overcome the recommended social distance several times.

**Figure 24:**
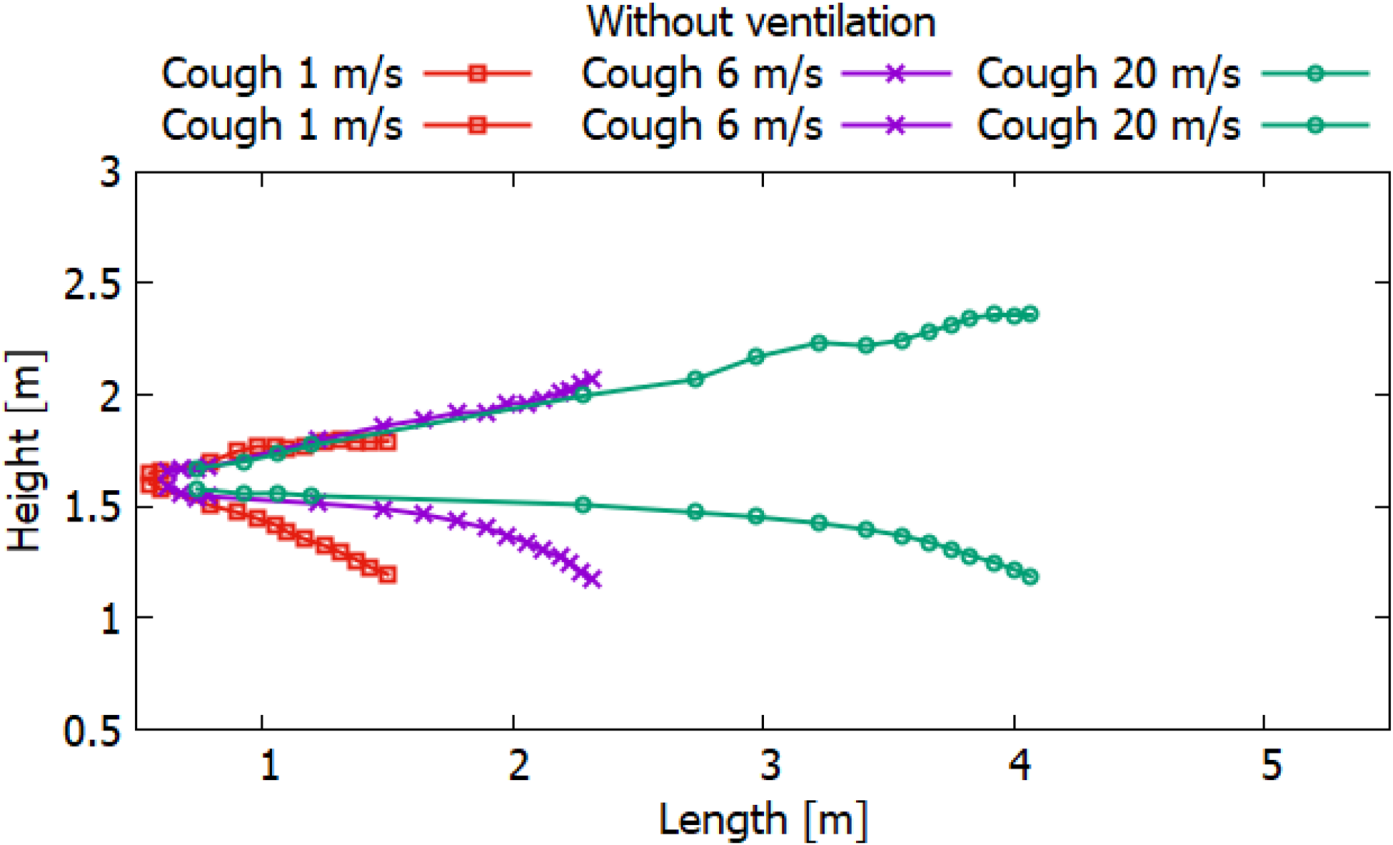
Particle propagation range for scenarios 1–3.

**Figure 25:**
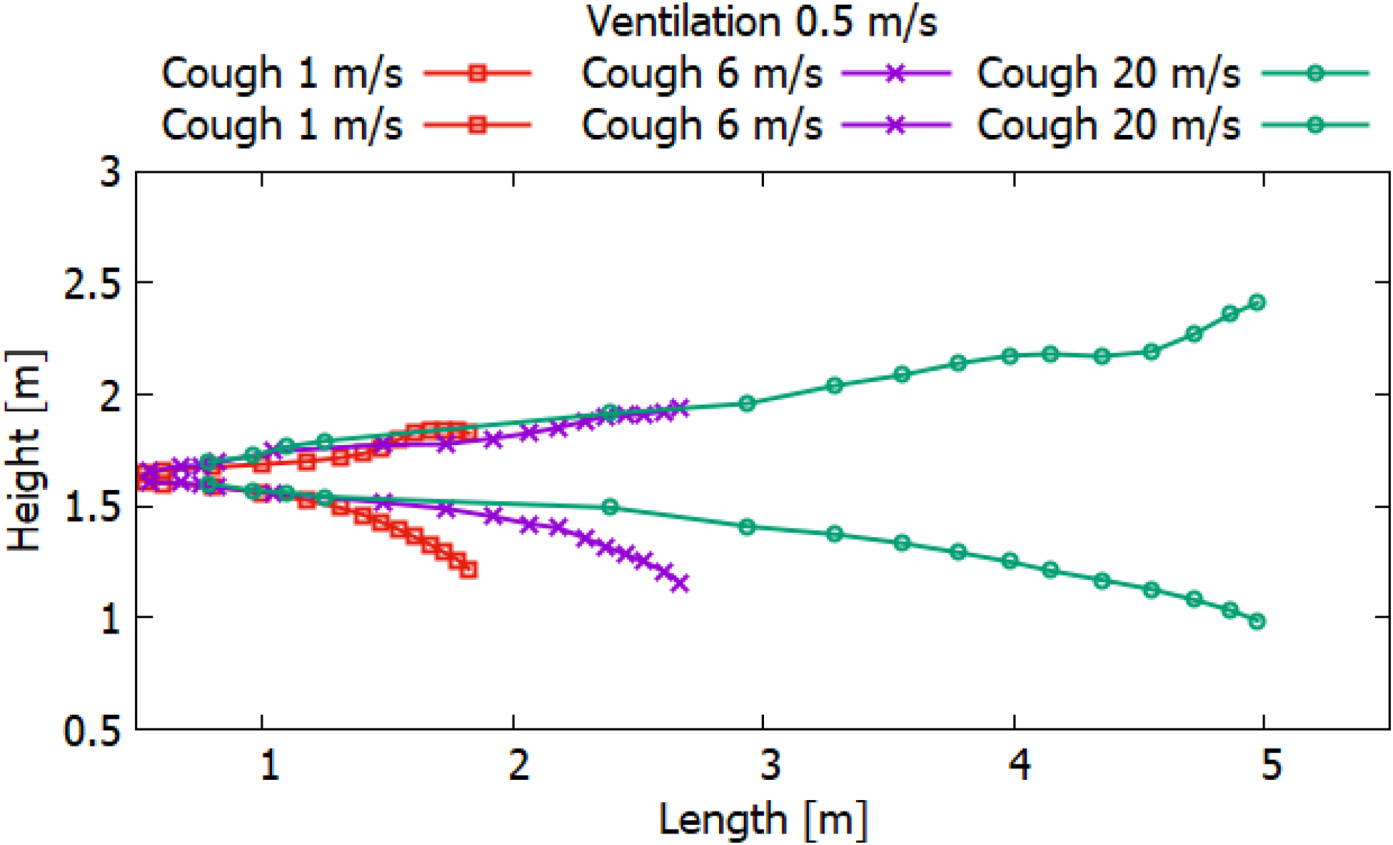
Particle propagation range for scenarios 4–6.

**Figure 26:**
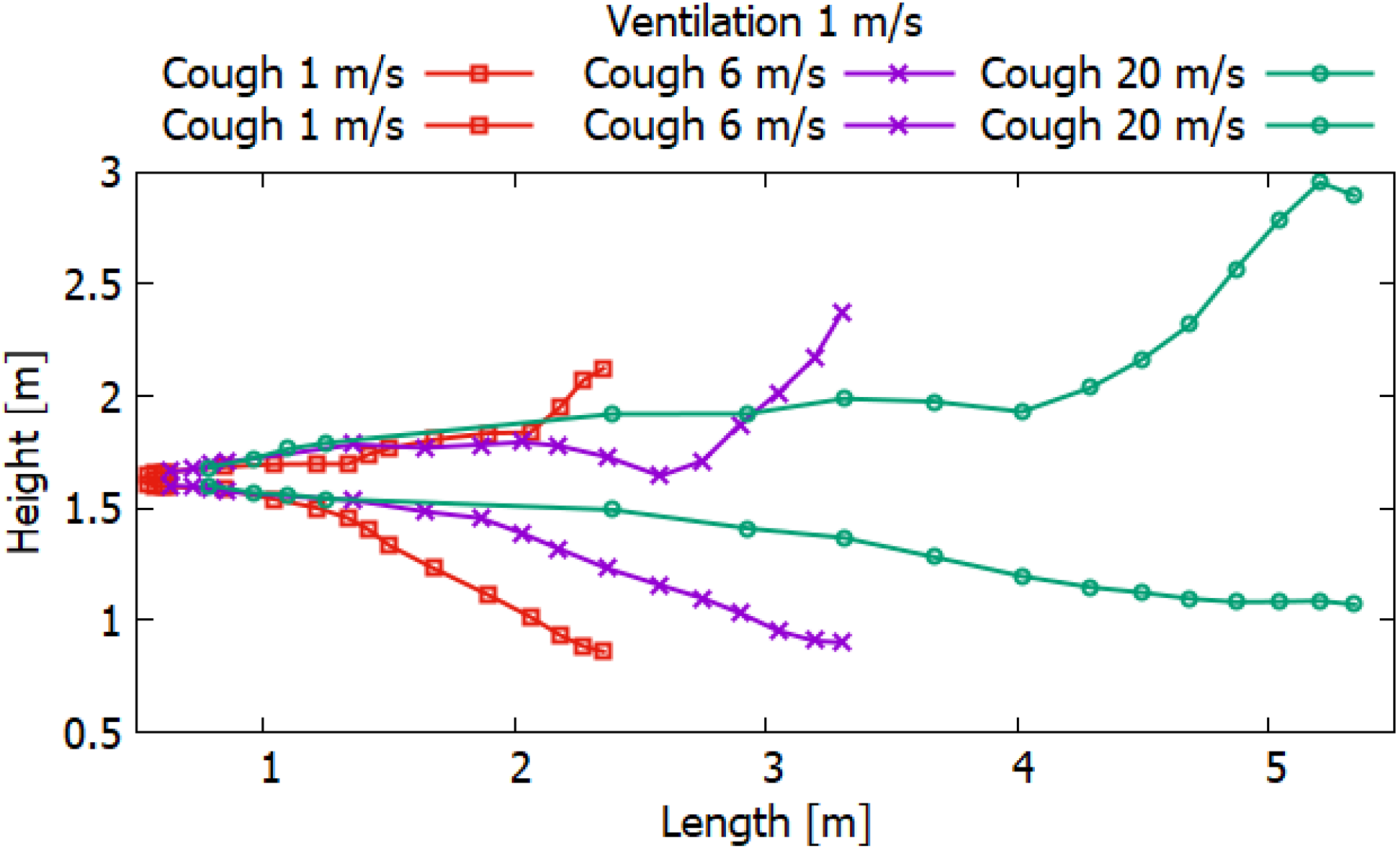
Particle propagation range for scenarios 7–9.

Particles can carry smaller viral particles and thus pose a greater hazard or risk in terms of human transmission of disease by airborne droplets. This study shows that when a person coughs, the rate of ventilation in the room significantly affects the transport of large particles. Without ventilation, large particles (10^–4^ to 10^–3^ m) settle to the ground a short distance from the person who is exhaling or coughing. However, their trajectory of more than 2 m will already be at an altitude well below 1.5 m. Thus, these droplets may not pose a hazard when in contact with adults at this 2 m distance. Small adults and children may be at greater risk if they are within the path of falling particles with large diameters. Figures 24, 25 and 26 show the results of particle propagation in space.

The results, presented in Fig. 24, show how particles spread in a room without ventilation. Different modes of particle ejection have a significant effect on particle length transport. In the mode of ejection of particles with a speed of 20 m/s, particles in 30 s can cover a distance 3.5 times more than when ejection with a velocity of 1 m/s and a distance 2 times more than when ejecting 6 m/s. However, the settling of particles with large sizes is approximately the same for all ejection modes. It should also be noted that the trajectory of an increase in the area of particle propagation without ventilation along the length and height tends to be linear. The results obtained for scenarios 4–6 presented in Fig. 25 also illustrate the linear behavior of the particle propagation trajectory along the length and height. It can be seen from these results that when ventilation is used in a room at a speed of 0.5 m/s, not only the distance along the length increases, but also the distance in height. It is worth noting a strong increase in the area of particle propagation in scenario 6 due to the increase in ejection and ventilation rates.

The results presented in Fig. 26 showed non-linear propagation of particles in the room, as there is a sharp increase in height. This dramatic increase is due to vortices that occur at the top of the room when ventilated at 1 m/s. As expected, the increase in the velocity of particle ejection increased the transport distance in length and height. However, it should be noted that with ventilation at a velocity of 1 m/s, the settling of particles in 40 s decreases with an increase in the ejection velocity. This phenomenon can be attributed to the prevailing momentum over gravity.

The obtained numerical results show that droplets or particles generated during normal respiration are transported over relatively short distances, while droplets or particles formed during coughing or sneezing can travel much longer, which can adversely affect protection the human body from infectious diseases. In many scenarios, particle transport exceeds the WHO social distance of 2 m. It should also be noted that different ventilation modes can greatly affect the area of particle propagation. From the obtained results, it should be noted that when recommending and choosing a social distance, not only the modes of emission of polluting particles, but also external conditions, especially momentum and gravity, should be taken into account.

## Conclusion

This work used CFD to investigate the transport and scattering of particles of various sizes (10^–4^ to 10^–6^) that occur when a person breathes, sneezes, or coughs. The process of emitting particles into the air was used to simulate a real human cough. Extensive computational studies have been carried out on the emission of particles from normal human breathing, sneezing and coughing. The validation of the ventilation model is in good agreement with the experimental data, which means that the entire mechanism can be efficiently modeled.

Numerical studies of the transport and distribution of particles or droplets formed during normal breathing, sneezing or coughing in a room lead to the following conclusions: in a normal breathing process, particles or droplets can only be transported over short distances; when sneezing or coughing, particles are transported over long distances. Sneezing or coughing at 20 m/s will cause particles or droplets to travel more than 3 m in 40 s. Sneezing propagation analysis showed a maximum impact zone of 4.84 m downstream, 1.13 m lateral and 1.82 m horizontal. Due to the difficult real conditions of the environment ventilation, it is necessary to take into account the social distance of more than 2 m, i.e. more than 5 m. It should also be noted that in the mode of particle ejection at a velocity of 20 m/s, particles in 30 s cover a distance 3.5 times more than when ejecting at a velocity of 1 m/s and the distance is 2 times greater than when ejecting 6 m/s. However, the sedimentation of particles for all modes of emission is approximately the same. So from the obtained numerical results, it can be noted that the social distance recommended by WHO of 2 m is performed for simple breathing for all cases (without ventilation and with ventilation). However, when coughing or sneezing, this distance is clearly not enough and it needs at least 5 m social distance in order not to get into the zone of exposure to these particles.

All results at high ejected velocities carry particles over a much greater distance due to the large amount of motion. This means that good personal habits, such as covering your nose and mouth when sneezing or coughing, or wearing a mask, are very important to prevent long-distance transport of particles, which in turn reduces the transmission of disease from person to person. It should be noted that the above results are based on simplified and ideal scenarios without considering many influencing factors such as temperature, humidity, evaporation of droplets and particles, etc. Therefore, the results should be used with care. However, this research can be seen as a direction in understanding the complex phenomena of particles transport of different sizes in rooms and, ultimately, in the prevention of transmission of infectious diseases.

## Data Availability

The datasets used and/or analyzed during the current study are available from the corresponding author on reasonable request.
